# Integrated single-nucleus transcriptomic and metabolomic insights into bud-to-leaf development and metabolite synthesis in tea plant

**DOI:** 10.1093/hr/uhaf281

**Published:** 2025-10-11

**Authors:** Xuecheng Zhao, Xiaoying Xu, Ning Chi, Yiming Liu, Xinxin Zhou, Jiqiang Jin, Chunlei Ma, Jianqiang Ma, Wei Chen, Mingzhe Yao, Liang Chen

**Affiliations:** State Key Laboratory of Tea Plant Germplasm Innovation and Resource Utilization, Tea Research Institute of the Chinese Academy of Agricultural Sciences, Hangzhou 310008, China; Yunnan Key Laboratory of Tea Germplasm Conservation and Utilization in the Lancang River Basin, West Yunnan University, No. 2, Xuefu Road, Lincang, Yunnan 677000, China; State Key Laboratory of Tea Plant Germplasm Innovation and Resource Utilization, Tea Research Institute of the Chinese Academy of Agricultural Sciences, Hangzhou 310008, China; Tea Research Institute, Zhejiang University, Hangzhou 310058, China; State Key Laboratory of Tea Plant Germplasm Innovation and Resource Utilization, Tea Research Institute of the Chinese Academy of Agricultural Sciences, Hangzhou 310008, China; Lc-Bio Technologies (Hangzhou) Co., Ltd, Hangzhou 310000, China; State Key Laboratory of Tea Plant Germplasm Innovation and Resource Utilization, Tea Research Institute of the Chinese Academy of Agricultural Sciences, Hangzhou 310008, China; State Key Laboratory of Tea Plant Germplasm Innovation and Resource Utilization, Tea Research Institute of the Chinese Academy of Agricultural Sciences, Hangzhou 310008, China; State Key Laboratory of Tea Plant Germplasm Innovation and Resource Utilization, Tea Research Institute of the Chinese Academy of Agricultural Sciences, Hangzhou 310008, China; National Key Laboratory of Crop Genetic Improvement and National Center of Plant Gene Research (Wuhan), Huazhong Agricultural University, Wuhan 430070, China; State Key Laboratory of Tea Plant Germplasm Innovation and Resource Utilization, Tea Research Institute of the Chinese Academy of Agricultural Sciences, Hangzhou 310008, China; State Key Laboratory of Tea Plant Germplasm Innovation and Resource Utilization, Tea Research Institute of the Chinese Academy of Agricultural Sciences, Hangzhou 310008, China; Yunnan Key Laboratory of Tea Germplasm Conservation and Utilization in the Lancang River Basin, West Yunnan University, No. 2, Xuefu Road, Lincang, Yunnan 677000, China

## Abstract

The tea plant is an important nonalcoholic beverage crop known for its abundant secondary metabolites, particularly in buds and leaves. However, the coordinated regulation of bud-to-leaf development and metabolism remains poorly understood. Here, we applied single-nucleus RNA sequencing (snRNA-Seq), bulk RNA sequencing (RNA-Seq), and metabolomics to comprehensively profile the developmental trajectory and metabolic characteristics of tea plant buds and leaves. The snRNA-Seq analysis revealed 17 cell clusters and 8 cell types in buds and leaves, respectively. Notably, the proportion of palisade mesophyll (PM) cells increased progressively during development, while proliferating cells (PC) decreased. Interestingly, key enzymes in the flavonoid biosynthetic pathway were specifically localized to PM cells. Metabolomic analyses demonstrated dynamic accumulation patterns of various metabolites, including phytohormones, flavonoids, and amino acids, as the buds transitioned to mature leaves. Using multi-omics profiling, we identified *CsmiRNA396b*, *CsUGT94P1*, *CsTCP3*, and *CsTCP14* as critical regulatory components. Enzyme activity assays confirmed that CsUGT94P1 catalyzes the conversion of flavonols into flavonol glycosides *in vitro*. Furthermore, *CsmiRNA396b* was found to regulate leaf development by inhibiting *CsGRF3* expression, while *CsTCP3* and *CsTCP14* played antagonistic roles in leaf development and flavonoid biosynthesis. Our findings provide novel insights into the regulatory mechanisms underlying bud-to-leaf development and metabolite production in tea plants.

## Introduction

Tea plants (*Camellia sinensis*) are perennial, evergreen woody plants whose buds and leaves are processed to produce tea, one of the most widely consumed nonalcoholic beverages worldwide [1, 2]. Tea quality and flavor are determined by the synthesis and accumulation of secondary metabolites in new shoots, including flavonoids, amino acids, alkaloids, and terpenoids [3]. While extensive research has been conducted on the biosynthesis and regulation of secondary metabolites, the integrated mechanisms coordinating bud-to-leaf development and metabolism remain largely unclear. Secondary metabolite synthesis in tea plants is tightly regulated by specific enzymes and transcription factors. For example, uridine diphosphate glycosyltransferases catalyze the glycosylation of flavonols and anthocyanins, influencing tea bitterness [4–6]. Transcription factors such as *CsMYB5*, *CsMYB12*, *CsMYB6*, and *CsMYB75* regulate the biosynthesis of catechins, flavonols, l-theanine, and anthocyanins, respectively [7–10]. Earlier studies, such as that by Yu *et al.* [11], elucidated regulatory networks controlling leaf and metabolite development in tea plants. For instance, *CsTCP3* and *CsTCP14* interact with the MBW complex to regulate catechin biosynthesis, while microRNAs (miRNAs), including *miR164b*, *miR319*, and *miR165*, were shown to influence shoot tip and leaf shape development [11]. Building on these findings, the present study integrates multiple omics approaches to further characterize the bud-to-leaf developmental trajectory and metabolic features.

Bud and leaf size are critical agronomic traits that influence both tea yield and quality. These traits are primarily governed by coordinated cell proliferation, growth, and differentiation, processes regulated by transcription factors such as the TEOSINTE BRANCHED 1 (*TB1*)/CYCLOIDEA (*CYC*)/PROLIFERATING CELL NUCLEAR ANTIGEN FACTOR 1 (*PCF1*) (*TCP*) family [12, 13]. *TCP* transcription factors are essential regulators of plant growth and development. For instance, *AtTCP3/4/10* negatively regulate *CUP-SHAPED COTYLEDON* (*CUC*) genes, which influence shoot meristem boundaries [14–16], while *TCP14* and *TCP15* modulate internode elongation and leaf morphology in *Arabidopsis thaliana* [17]. Moreover, *AtTCP3* interacts with MYB transcription factors to promote flavonoid biosynthesis [18].

miRNAs are regulatory RNAs that play diverse roles in plant development [19, 20]. In leaf development, miRNAs including *miR319*, *miR164*, *miR396*, and *miR156/172* regulate their respective target genes: *TCP3* (regulated by *miR319*), *CUC* (by *miR164*), growth-regulating factors (*GRFs*, by *miR396*), and SQUAMOSA PROMOTER BINDING PROTEIN-LIKE (*SPLs*, by *miR156/172*). Specifically, *miR396* targets GRF genes to control leaf size [15, 16, 21, 22]. These miRNAs often function alongside phytohormones, key signaling molecules that coordinate bud and leaf development through gene regulation [23]. Auxin (AUX), cytokinin (CK), abscisic acid (ABA), gibberellic acid (GA), ethylene (ET), jasmonic acid (JA), salicylic acid (SA), and brassinolide (BR) are particularly prominent regulators [22]. Furthermore, miRNAs can integrate into hormone pathways. For instance, *miR396* negatively regulates *GRF* expression, affecting the biosynthesis of ABA, GA, and AUX [24]. Other miRNAs, such as *miR160*, *miR167*, *miR156*, and *miR319*, similarly regulate AUX, CK, and GA biosynthesis, modulating responses to JA and ABA throughout plant development [25–27].

Advanced omics technologies, including single-cell RNA sequencing (scRNA-seq) and snRNA-seq, have been successfully applied to resolve cell-specific questions in plants. For example, Wang *et al.* identified marker genes in different cell types of tea plant leaves, while Lin *et al.* explored the molecular mechanisms underlying theanine biosynthesis in tea roots using scRNA-seq [28, 29]. Hu *et al.* further revealed cell type-specific gene expression and regulatory networks in tea root tips via snRNA-seq [30]. Here, we employed snRNA-seq, bulk RNA-seq, and metabolomics to elucidate the regulatory mechanisms and metabolic dynamics during bud-to-leaf development in tea plants. The snRNA-seq results identified 8 major cell types and 17 cell clusters in buds and leaves. Enzyme activity assays confirmed the role of CsUGT94P1 in catalyzing flavonol glycoside formation, while *CsmiRNA396b* regulated leaf size. Additionally, *CsTCP3* and *CsTCP14* were shown to control leaf development and flavonoid biosynthesis. These findings shed light on the molecular processes driving bud-to-leaf development and secondary metabolite synthesis in tea plants and provide a framework for future research in related plant systems.

## Results

### Sequencing statistics and cell type identification

**Figure 1:**
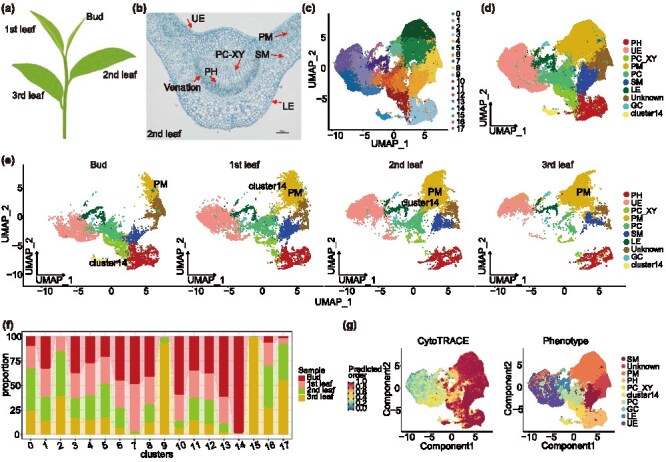
Atlas of cell types of different developing tissues of tea plant. (a) Tender new growth of tea plant, including bud, first leaf, second leaf, and third leaf. (b) Mid-vein cross-section of the second leaf. Bar = 100 μm. (c) Visualization of 17 cell clusters using UMAP, with each dot representing a single cell and colors indicating cell clusters. (d) Visualization of eight cell types and cluster 14 using UMAP. Dots, individual cells; colors represent different cell types. (e) Cell type distribution in different tissues of tea plants. (f) Cell cluster distribution in different tissues. (g) Stemness analysis of different cell types. PM: 0, 5, 9, and 15; UE: 1, 2, 7, and 13; PH: 3; SM: 4, PC: 6 and 12; unknown: 8; PC-XY: 10; LE: 11 and 17; GC: 16; cluster 14.

Tea plant’s new shoots, including the bud, first leaf, second leaf, and third leaf, exhibit unique quality components and developmental characteristics. To investigate these features, we performed snRNA-Seq on the apical bud and sequential leaves (Fig. 1a). Tissue sections of the first, second, and third leaves revealed the presence of upper epidermal (UE), lower epidermal (LE), palisade mesophyll (PM), proliferating xylem (PC-XY), phloem cells (PH), and spongy mesophyll (SM) (Fig. 1b, Fig. S1a–d). After snRNA-Seq data processing, a total of 48 047 nuclei were obtained, ranging from 9857 to 13,645 nuclei per sample. The median gene count ranged from 1667 to 2031. Following quality filtering, 8855 to 12 179 high-quality nuclei per sample were retained (Supplemental Data 1). Dimensionality reduction clustering analysis identified 17 distinct clusters, which were assigned to 8 cell types based on marker genes: PM, UE, LE, PC-XY, PH, SM, proliferating cells (PC), and guard cells (GC) (Fig. 1c and d, Fig. S2a and b, Supplemental Data 2). Functional annotation of marker genes for different cell types is in Supplemental Data 3. Notably, the proportion of PM cells increased progressively from bud to leaf development, while the proportion of PC decreased (Fig. 1e, Fig. S3a, Supplemental Data 4). Analysis of the 17 clusters revealed developmental-specific changes, particularly in cluster 14, which was predominantly distributed in the bud (Fig. 1f, Fig. S3b, Supplemental Data 5 and 6). Cluster 14 was divided into six subclusters based on marker gene expression (Fig. S4a and b). These subclusters were predominantly found in the bud, with cluster 3 appearing in the first leaf, cluster 1 in the second leaf, and no clusters detected in the third leaf (Fig. S4c). Hierarchical Weighted Gene Co-expression Network Analysis (hdWGCNA) of cluster 14 revealed four primary modules of gene co-expression (Fig. S4d, Supplemental Data 7 and 8). KEGG enrichment analysis showed that genes in cluster 14 were associated with key metabolic processes, including protein homodimerization, cytosolic processes, multicellular organism development, nucleic acid binding, and response to karrikin (Fig. S4e, Supplemental Data 9). These findings suggest that cluster 14 plays a critical role in promoting cellular development in the bud. Additionally, KEGG enrichment analysis showed that each cell type exhibited distinct metabolic characteristics. For instance, SM cells were enriched for pathways related to photosynthesis and starch and sucrose metabolism, while PC cells exhibited enrichment for ribosome biosynthesis (Fig. S5). To explore the differentiation potential of each cell type, Cyto-TRACE analysis was performed, and results indicated higher differentiation potential in SM and PM cells compared to UE and LE cells (Fig. 1g, Fig. S6a and b). Furthermore, differentiation trajectories of various cell types within each tissue were mapped (Fig. S6c). Collectively, these results demonstrate that cell type composition and functional characteristics dynamically shift during bud-to-leaf development.

### Differentiation trajectories in the development of PM and PC

**Figure 2:**
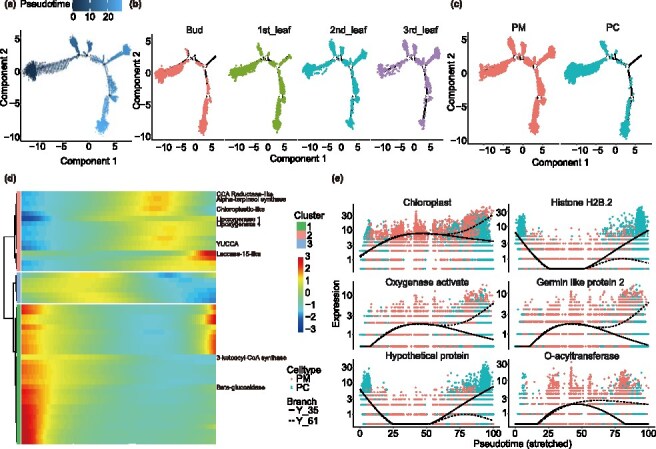
Differentiation trajectory and cell fate decisions analyzed by pseudo-time analysis. (a) Pseudo-time model graph. (b and c) Ordering of cells along the differentiation trajectory, presented by pseudo-time states, samples, and cell types. (d) Heatmap showing the top 50 significantly changed genes in PM and PC cell types. (e) Six functional genes with the most significant changes identified at the fourth branch.

To further validate the identified cell types and investigate the continuous differentiation trajectories during bud-to-leaf development, we performed pseudo-time analysis using partially clustered cells (Fig. 2). Specifically, PM cell abundance increased progressively, while PC cell abundance decreased (Fig. 1e). Pseudo-time trajectory analysis, conducted using the Monocle 2 package, revealed four key branch points, corresponding to distinct cellular differentiation states (Fig. 2a and b). At early developmental stages, buds showed initial differentiation patterns, with PM and PC exhibiting typical changes (Fig. 2c, Fig. S7a). Key genes associated with PM and PC differentiation were identified through branch-specific analysis. Among the top 50 genes, pathways related to phenylpropanoid biosynthesis, monoterpenoid synthesis, photosynthesis, alpha-linolenic acid metabolism, and fatty acid elongation were enriched (Fig. 2d). Further analysis of branch node 4 revealed significant changes in the top 100 genes, which were primarily involved in chloroplast biogenesis and photosynthesis (Fig. S7b). Branched expression analysis highlighted the top 6 significantly altered genes, which were enriched for processes including chloroplast formation, suberin biosynthesis, and wax biosynthesis (Fig. 2e). These results demonstrate that bud-to-leaf development is marked by dynamic shifts in cell types, with PM cells progressively adapting to photosynthetic functions and PC cells diminishing as tissues mature. This differentiation trajectory reflects the adaptive changes necessary for tissue survival and functionality during leaf development.

### Metabolic patterns of phytohormones in different tissues

**Figure 3:**
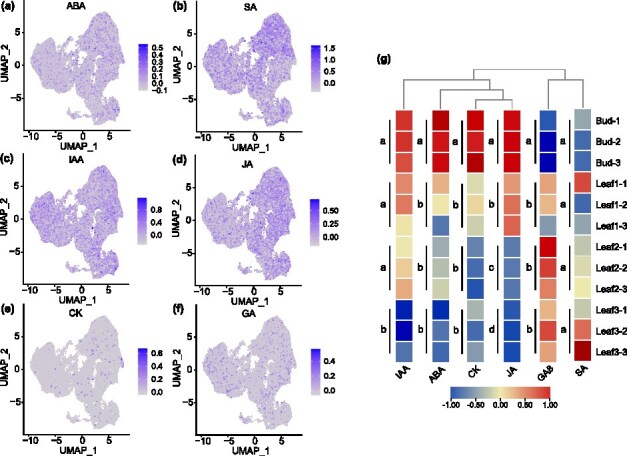
Patterns of hormone accumulation during the development of tea bud and leaf. (a–f) Visual analysis of expression patterns of six types of hormone-related synthetic genes. (g) Accumulation analysis of six types of hormones at different stages of bud and leaf development. Two-factor ANOVA was performed, and the differences were analyzed using the Tukey test.

Phytohormones are essential regulators of plant growth and development, orchestrating processes such as seed dormancy, vegetative growth, leaf and flower formation, fertilization, and fruit development [31]. Different types of phytohormones often interact antagonistically or synergistically to regulate these stages. To elucidate the synthesis and accumulation patterns of phytohormones during the bud-to-leaf developmental trajectory in tea plants, we employed a multi-omics approach. Visualization of phytohormone synthases revealed differential expression patterns among six major phytohormone classes (Fig. 3). Specifically, genes encoding synthases for SA and JA were highly expressed, with SA and JA synthases predominantly localized in PM cells (Fig. 3b and d, Supplemental Data 10). In contrast, synthases for ABA, CK, and GA exhibited lower expression levels and a more scattered distribution (Fig. 3a, e, and  f). The expression of IAA synthetase was evenly distributed in every cell type (Fig. 3c). Additionally, JA accumulation declined across the bud, first leaf, second leaf, and third leaf (Fig. 3g). A similar downward trend was observed for IAA, ABA, and CK accumulation during tissue development (Fig. 3g). In contrast, GA8 exhibited increased accumulation with tissue maturation (Fig. 3g, Supplemental Data 11). These results indicate that phytohormone accumulation patterns are distinct during different stages of bud-to-leaf development. Early developmental stages are primarily regulated by auxin and cytokinin to promote cell division and differentiation, while gibberellins facilitate growth in later stages. This functional specialization highlights the diverse roles of phytohormones in tissue development and maturation.

### MicroRNAs regulate leaf development

**Figure 4:**
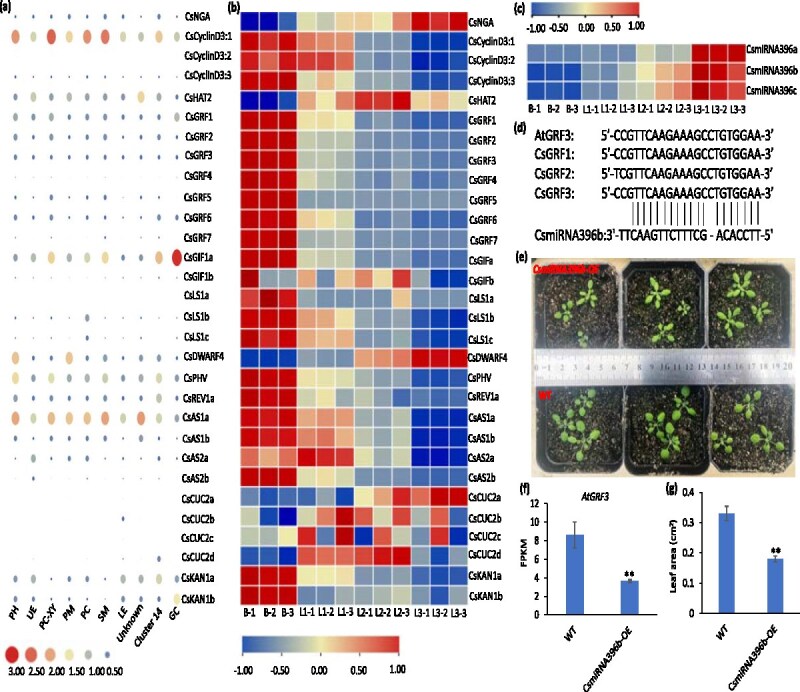
Regulatory mechanisms controlling tea leaf development. (a and b) Visual analysis of expression patterns of leaf regulatory genes in different tissues and cell types. (c) Expression analysis of *CsmiRNA396a/b/c* in bud and leaf tissues. (d) Comparative analysis of *AtGRF3* and *CsGRF1/2/3* and *CsmiRNA396b*. (e) Phenotypic analysis of *A. thaliana* overexpressing *CsmiRNA396b*. (f) Expression pattern analysis of *AtGRF3* in wild-type (WT) and *CsmiRNA396b* overexpressed lines. (g) Area of fully expanded first leaves of wild-type and *CsmiRNA396b* overexpressed lines. Significant differences were performed by Student *t* test (*P < .05 and **P < .01). B: bud; L1: first leaf; L2: second leaf; L3: third leaf.

To investigate regulatory factors influencing bud-to-leaf development, we analyzed gene expression patterns of key regulatory pathways using snRNA-seq and RNA-seq. Genes involved in cell division and leaf development, such as TCPs, GRFs, GRF-interacting factors (GIFs), and CUCs, were visualized to understand their dynamic expression patterns [14, 16, 32]. Visual analysis of different types of developmental regulatory genes showed that *CsCyclinD3:1, CsGIF1a*, and *CsAS1a* were more highly expressed in different types of cells than other genes (Fig. 4a). Notably, the expression levels of genes *CsNGA*, *CsHAT2*, and *CsDWF4* in PM are all higher than those in PC cells, and the expression levels of these genes continue to increase with the development of the leaves (Fig. 4a and b, Supplemental Data 12). Expression analysis revealed that *CsGRFs* exhibited decreasing expression levels as leaves developed (Fig. 4b, Supplemental Data 12). The miRNAs are important post-transcriptional regulators of gene expression that control developmental processes, including leaf formation. Transcriptome sequencing identified several miRNAs with dynamic expression patterns during bud-to-leaf development. Interestingly, the expression levels of *CsmiRNA156s*, *CsmiRNA164s*, and *CsmiRNA396s* increased with tissue development, whereas *CsmiRNA319s* showed a decline (Fig. S8a, Supplemental Data 13). However, *CsmiRNA166s* exhibited inconsistent expression patterns across tissues, suggesting functional diversity among its isoforms (Fig. S8a). To identify key miRNAs involved in leaf development, we conducted further analysis and highlighted critical miRNA-target pairs (Fig. 4c and d, Fig. S8b). For example, transgenic studies revealed that *CsmiRNA396b* inhibited leaf blade size and weight in *Arabidopsis* by targeting *AtGRF3* expression (Fig. 4e–g, Fig. S8c). This outcome is attributed to *miR396* acting as a negative regulator of mitotic cell division through affecting *AtGRF* expression. Furthermore, *GRF* also modulates GA and CK pathways as both positive and negative regulators [24, 33]. These findings demonstrate that miRNAs play a pivotal role in the developmental trajectory of tea plant buds and leaves by targeting specific regulatory genes. The interplay between miRNAs and their targets fine-tunes key processes such as cell division, differentiation, and hormone signaling, ultimately influencing leaf size and tea quality.

### Changes in metabolites during bud to leaf development

**Figure 5:**
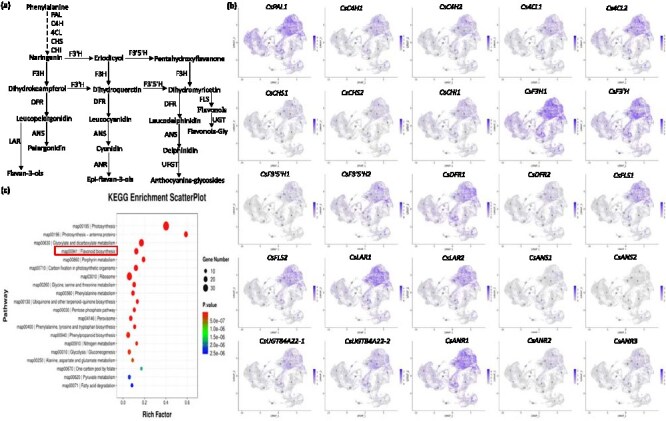
Visualization of flavonoid metabolic flow. (a) Flavonoid biosynthesis model in tea plant. (b) Visual analysis of genes associated with flavonoid synthesis pathway in tea plant bud and leaf. (c) KEGG enrichment analysis of PM cell type.

Tea plant new shoots are rich in secondary metabolites, particularly flavonoids, which play a critical role in determining tea quality [3]. Flavonoids are synthesized through the phenylpropanoid pathway via a series of enzymatic reactions (Fig. 5a). Visualization of snRNA-Seq data showed that the majority of flavonoid biosynthetic enzymes are highly accumulated in PM cells (Fig. 5b, Fig. S9, Supplemental Data 14), and KEGG enrichment analysis showed PM cells were enriched for pathways related to flavonoid biosynthesis (Fig. 5c). Metabolite profiling demonstrated that most flavonoids accumulated progressively with tissue development, and among these, flavonol glycoside is particularly obvious (Fig. S10a, Supplemental Data 15). Additionally, six major classes of catechins were detected; among them, the increasing trends of C and ECG are the most pronounced (Fig. S10b). Anthocyanins were primarily present as glycosides, including cyanidin 3-*O*-rutinoside, cyanidin 3,5-*O*-diglucoside, and delphinidin 3-*O*-glucoside, among others (Fig. S10c). Expression levels of key flavonoid biosynthetic genes mirrored the metabolite accumulation patterns, increasing in concert with tissue development, such as *CsC4H*, *CsLAR*, and *CsUGT84A22* (Fig. S10d). In contrast to flavonoids, the majority of amino acids, especially l-theanine accumulation, was highest in the bud and declined during tissue maturation, consistent with decreasing expression levels of amino acid biosynthetic genes (Fig. S11a–c, Supplemental Data 16 and 17). Additionally, chlorophyll biosynthesis genes showed progressive upregulation across tissues (Fig. S12, Supplemental Data 18). Notably, chlorophyll biosynthetic genes were also concentrated in PM cells, aligning with their key metabolic role in photosynthesis (Fig. 5c). These results highlight the differential synthesis and accumulation patterns of metabolites during bud-to-leaf development. Flavonoid accumulation increases in mature tissues, providing defense against abiotic stress, while chlorophyll production rises to support photosynthetic function.

### CsTCP3 and CsTCP14 act in an antagonistic manner to regulate leaf development and flavonoids

Metabolomic analysis revealed significant differences in flavonoid glycosides accumulation across tissues, such as luteolin 7-*O*-glucoside, quercetin 3-*O*-glucoside, and kaempferol 3-*O*-galactoside (Fig. S10a). This result indirectly indicates that during bud and leaf development, the content of flavonoid glycosides is an accompanying phenotype, and the major gene family driving the variation in the levels of this substance is the glycosyltransferase gene family. Here, we identified a *CsUGT94P1* whose expression coincided with flavonoid glycoside accumulation, based on snRNA-Seq, RNA-Seq, and metabolomic data (Fig. S13a–c, Supplemental Data 19). Enzyme activity assays demonstrated that CsUGT94P1 could catalyze the formation of kaempferol-3-*O*-glucoside or quercetin-3-*O*-glucoside from kaempferol or quercetin using UDP-glucose as a donor *in vitro* (Fig. S13d and e). This result indicates that flavonoid glycoside content continuously increases during bud and leaf development, contributing to the characteristic bitterness and astringency of tea. This accumulation is driven by the high expression of *UGTs* in buds and leaves, which catalyze the glycosylation of flavonoids as tissues mature [34].

**Figure 6:**
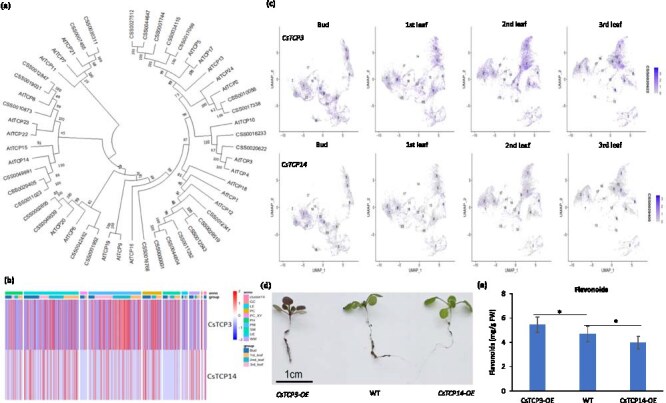
Accumulation patterns and formation mechanisms of flavonoids. (a) Phylogenetic tree analysis of TCPs transcription factors in tea plant and *A. thaliana*. (b and c) Expression patterns of *CsTCP3* and *CsTCP14* transcription factors in different tissue, note: (wm: unknown). (d and e) Phenotypic analysis of *CsTCP3* and *CsTCP14* overexpression in *A. thaliana* and corresponding flavonoids content analysis. Data were from three independent experiments and expressed as means ± SD (*n* = 3). The differences were analyzed in two tailed comparisons with the control, and *P <0 .05, **P <0 .01 in Student *t* test.

In addition to this, we identified two TCP transcription factors, *CsTCP3* and *CsTCP14*. The TCP family of transcription factors is divided into two classes: class I TCPs, which promote tissue development, and class II TCPs, which act as repressors of cell division [11, 35]. *CsTCP14* was classified as a class I TCP, while *CsTCP3* belonged to class II (Fig. 6a) [11]. Our combined snRNA-Seq and RNA-Seq analysis revealed that *CsTCP3* and *CsTCP14* exhibited opposing expression patterns during tissue development (Fig. S14a and b). *CsTCP3* expression increased with tissue maturation and was predominantly localized in PM cells (Fig. 6b and c), correlating positively with flavonoid accumulation. In contrast, *CsTCP14* showed decreasing expression during development. Furthermore, transgenic plants further validated these results: *CsTCP3-OE* enhanced flavonoids accumulation, while *CsTCP14* overexpression inhibited it (Fig. 6d and e). In addition to metabolic changes, significant differences in leaf phenotypes were observed in transgenic plants. Compared to the wild type, *CsTCP3* overexpression suppressed leaf size and reduced leaf margin serration. Conversely, *CsTCP14* overexpression promoted leaf enlargement and suppressed serrations (Fig. S14c). Additionally, functional analysis demonstrated that inhibition of *CsTCP3* expression led to downregulation of *CsUGT94P1* and a corresponding reduction in flavonol glycoside content (Fig. S14d). In conclusion, *CsTCP3* and *CsTCP14* play antagonistic roles in regulating flavonoid biosynthesis and leaf development. *CsTCP3* promotes flavonoid accumulation and suppresses leaf size, and reduces leaf margin serration, while *CsTCP14* inhibits flavonoid biosynthesis and promotes leaf growth. These findings provide critical insights into the functional diversity of TCP transcription factors in tea plant development and secondary metabolite regulation.

## Discussion

### Application of multi-omics in tea plant research

**Figure 7:**
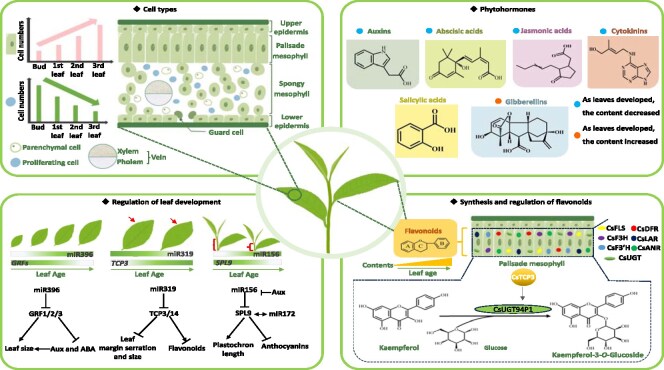
Model for the coordinated regulation of bud-to-leaf development and metabolism in tea plant. *Cell types:* bud and leaf contain eight cell types, of which PM is increasing with development, while PC cell number is decreasing with development; *phytohormones**:*** the pattern of phytohormone accumulation varied in different tissues, where IAA, ABA, JA, and CK decreased with tissue development, while GA8 increased with tissue development; *regulation of leaf development: CsmiRNA396* regulates leaf size by repressing the expression of *GRF1/2/3*, while *CsmiRNA319* can interfere with the expression of *CsTCP3/CsTCP14* to regulate leaf margin serration, leaf size, and flavonoid biosynthesis. *CsmiRNA156* regulates plastochron length and anthocyanin synthesis by repressing *CsSPL9* expression; *synthesis and regulation of flavonoids:* flavonoids increase with tissue development, and the major flavonoid synthases are concentrated in the PM. CsTCP3 promotes the accumulation of flavonol glycosides by regulating the expression of *CsUGT94P1*.

With the advancement of technology, multi-omics approaches, including genomics, metabolomics, RNA-Seq, scRNA-Seq, and snRNA-Seq, have emerged as powerful tools in plant research. Recent applications of these technologies in plants such as cotton, rice, Chinese cabbage, *Catharanthus roseus*, and tea plants have resolved numerous scientific questions at the cellular level [30, 36–39]. In this study, we combined snRNA-Seq, RNA-Seq, and metabolomics to elucidate the bud-to-leaf developmental trajectory and associated metabolic changes in tea plants. Seventeen distinct clusters and eight cell types were identified based on marker gene expression (Figs 1c, d and 7). Cluster 14, predominantly localized in the bud, was further subdivided into six subclusters, all of which were restricted to the bud (Fig. S4c). KEGG analysis indicated that cluster 14 primarily regulated embryonic development, chloroplast biogenesis, and cytoplasmic processes (Fig. S4e). Cell stemness analysis demonstrated that SM and PM cells exhibited higher stemness compared to epidermal cells, which showed lower stemness (Fig. S6). Pseudo-time analysis further revealed differentiation characteristics of PM and PC cells, showing that PM increased while PC decreased during tissue maturation. The differentiation at node 4 was driven by a suite of developmental genes (Figs 1e and 2d). Integrating snRNA-Seq, RNA-Seq, and metabolomic data allowed us to identify key regulators of leaf development and metabolite synthesis, including *CsmiRNA396*, which regulates leaf size; CsUGT94P1, responsible for flavonoid glycoside biosynthesis; and CsTCP3 and CsTCP14, which regulate leaf development and flavonoid synthesis.

### Dynamic changes in metabolite accumulation during tea plant bud-to-leaf development

In tea plant germplasm resources, metabolite content varies significantly across different varieties and tissues at various developmental stages [40, 41]. To better understand these dynamics, we analyzed the metabolite accumulation patterns using metabolomic techniques. Our analysis revealed that the accumulation of IAA, JA, ABA, and CK decreased as tissues matured. In contrast, GA8 showed increasing accumulation with bud-to-leaf development (Fig. 3). These trends reflect the distinct roles of phytohormones at different developmental stages. For instance, IAA promotes early tissue development, including bud formation and leaf elongation, while JA primarily mediates responses to biotic stress during later stages. In addition to phytohormones, chlorophyll content was closely monitored, as its synthesis is vital for plant growth and energy production. Chlorophyll accumulation increased progressively with tissue development, corresponding with increased expression of chlorophyll biosynthetic genes (Fig. S12). This trend highlights the importance of chlorophyll in photosynthetic function as tissues mature. Unlike phytohormones and chlorophyll, flavonoids represent important secondary metabolites that significantly influence tea quality. Here, metabolomics analysis demonstrated that flavonoid accumulation, particularly flavonol glycosides, anthocyanin glycosides, and catechins, consistently increased during bud-to-leaf development (Fig. S10). Visualization of gene expression confirmed that the major flavonoid biosynthetic genes showed a continuous upregulation in tandem with flavonoid accumulation (Fig. S10d). In contrast, amino acids, particularly l-theanine, exhibited an opposite trend, with higher accumulation in the bud and a decline during leaf development (Fig. S11). This discrepancy reflects the functional specialization of metabolites: phytohormones regulate bud and leaf development, chlorophyll supports photosynthesis, and flavonoids provide defense against environmental stresses, while amino acids contribute to early-stage growth and metabolic activity.

### Synergistic regulation of bud-to-leaf development by miRNAs, phytohormones, and TFs in tea plant

Bud-to-leaf development is a highly regulated process influenced by multiple factors, including phytohormones, microRNAs, transcription factors (TFs), and environmental cues. The findings of this study highlight the complex regulatory networks that orchestrate bud and leaf development, with miRNAs, TCPs, and phytohormones acting in concert. Numerous studies have established the role of miRNAs in regulating bud and leaf development. For instance, *miRNA319* regulates leaf extension and leaf size by repressing *TCP3/4/5/14*, transcription factors that also participate in JA biosynthesis [16, 42]. Similarly, *miRNA396* promotes leaf size by targeting *GRFs*, which are regulators of AUX, ABA, and GA biosynthesis [21, 24]. Expression pattern analysis revealed that Cs*miRNA156*, Cs*miRNA166*, and Cs*miRNA396* showed increasing expression as tissues developed, while *miRNA319* expression gradually declined, and this result shows that miRNAs with different functions exist in tea plants (Fig. S8a). Further investigation identified five miRNA-target pairs that regulate bud-to-leaf development (Fig. S8b). For example, *CsmiRNA396* regulates leaf size through its target genes *CsGRF1/2/3*, a relationship that was particularly evident in this study (Figs 4d and 7). The other four miRNA-target combinations include *CsmiRNA156a* and *CsSPL9*, *CsmiRNA164a* and *CsCUC2b*, *CsmiRNA166a* and *CsREV1*, and *CsmiRNA319a* and *CsTCP3*. In these regulatory relationships, *CsmiRNA156* modulates plastochron length and anthocyanin synthesis by repressing *CsSPL9* expression [43, 44]. The mechanisms by which the remaining miRNAs and their target genes regulate leaf development and metabolite synthesis have been elaborated in previous studies [11]. These regulatory interactions reveal a coordinated network where miRNAs, their target genes, and phytohormones interact synergistically to fine-tune bud and leaf development. In summary, the bud-to-leaf developmental process is regulated not by a single factor but through a complex interplay between phytohormones, miRNAs, and transcription factors. This study provides a comprehensive understanding of how miRNA-target networks, in combination with hormonal pathways, control leaf size, developmental timing, and metabolite accumulation in tea plant.

## Materials and methods

### Plant materials

Five-year-old tea plants (‘Shuchazao’) were cultivated at the experimental tea garden of the Tea Research Institute, China Academy of Agricultural Sciences in Hangzhou, Zhejiang Province, China (latitude 30.1807°N, longitude 120°9′58″E). During April 2023, we collected samples from four developmental stages: bud, first leaf, second leaf, and third leaf.

### Metabolite extraction and detection

Flavonoids and amino acids were extracted from bud, first leaf, second leaf, and third leaf samples following our previously established protocol [3]. Phytohormones were similarly extracted from these tissues and analyzed by UHPLC–MS/MS using a 7500 Triple Quadrupole mass spectrometer (AB Sciex, USA), with methods adapted from Yin *et al.* [45].

### Paraffin sectioning

The first leaf, second leaf, and third leaf of tea plants were selected as the paraffin section sample, and detailed description is provided in Methods S1.

The extraction of single nuclei and the associated analysis methods are described in detail in Methods S1.

#### Ectopic expression of *CsmiRNA396b, CsTCP3* and *CsTCP14* in *Arabidopsis*

The ORFs of *CsmiRNA396b*, *CsTCP3*, and *CsTCP14* were ligated into the entry vector pDONR221 using Gateway BP Enzyme mix (Invitrogen, Carlsbad, CA, USA). Following sequence verification, these were transferred to the destination vector PB2GW7 via LR recombination. The constructs were then electroporated into *Agrobacterium* GV3101 and introduced into *Arabidopsis* through floral dip transformation. All primer sequences are provided in Supplemental Data 20.

#### Enzymatic assays of recombinant CsUGT94P1 *in vitro*

To investigate the enzymatic activity of CsUGT94P1, we expressed the gene in *Escherichia coli* with the constructed vector pMAL-c5X-CsUGT94P1, and the detailed experimental procedure and operation refer to the preliminary experimental scheme of this research group [6, 46].

#### Suppression of CsTCP3 in tea shoot tips by using candidate antisense oligonucleotides

The asODN targeting CsTCP3 was designed using Soligo software (http://sfold.wadsworth.org/cgi-bin/soligo.pl). For gene silencing, fresh shoot tips (apical bud and first leaf) of tea cultivar ‘SCZ’ were treated with 40 μM asODN-CsTCP3 in 2-ml Eppendorf tubes for varying durations. Controls were incubated with sense oligonucleotides (sODN) at the same concentration. Samples were collected at designated time points for RNA extraction and flavonol quantification.

### Statistical analysis

Single-nucleus RNA sequencing analysis was performed using Omi Studio tools (https://www.omicstudio.cn/tool/). Cell types were visualized through tSNE and UMAP projections, while gene expression patterns were displayed using marker gene dot plots and hierarchically clustered heatmaps. All data were normalized by Z-score transformation prior to Euclidean distance-based clustering.

## Supplementary Material

Web_Material_uhaf281
