# Supplementary material for: Integrated single-nucleus transcriptomic and metabolomic insights into bud-to-leaf development and metabolite synthesis in tea plant
Source: Hortic Res. 2025 Oct 11;13(1):uhaf281. doi: 10.1093/hr/uhaf281 (PMC12871078; doi:10.1093/hr/uhaf281)
Supplement: Web_Material_uhaf281 [file web_material_uhaf281.zip › Reviesd Supplemental information.pdf]

Integrated single-nucleus transcriptomic and metabolomic insights into bud-to-leaf development and metabolite synthesis in tea plant (*Camellia sinensis*)

Supporting Information

Figure S1

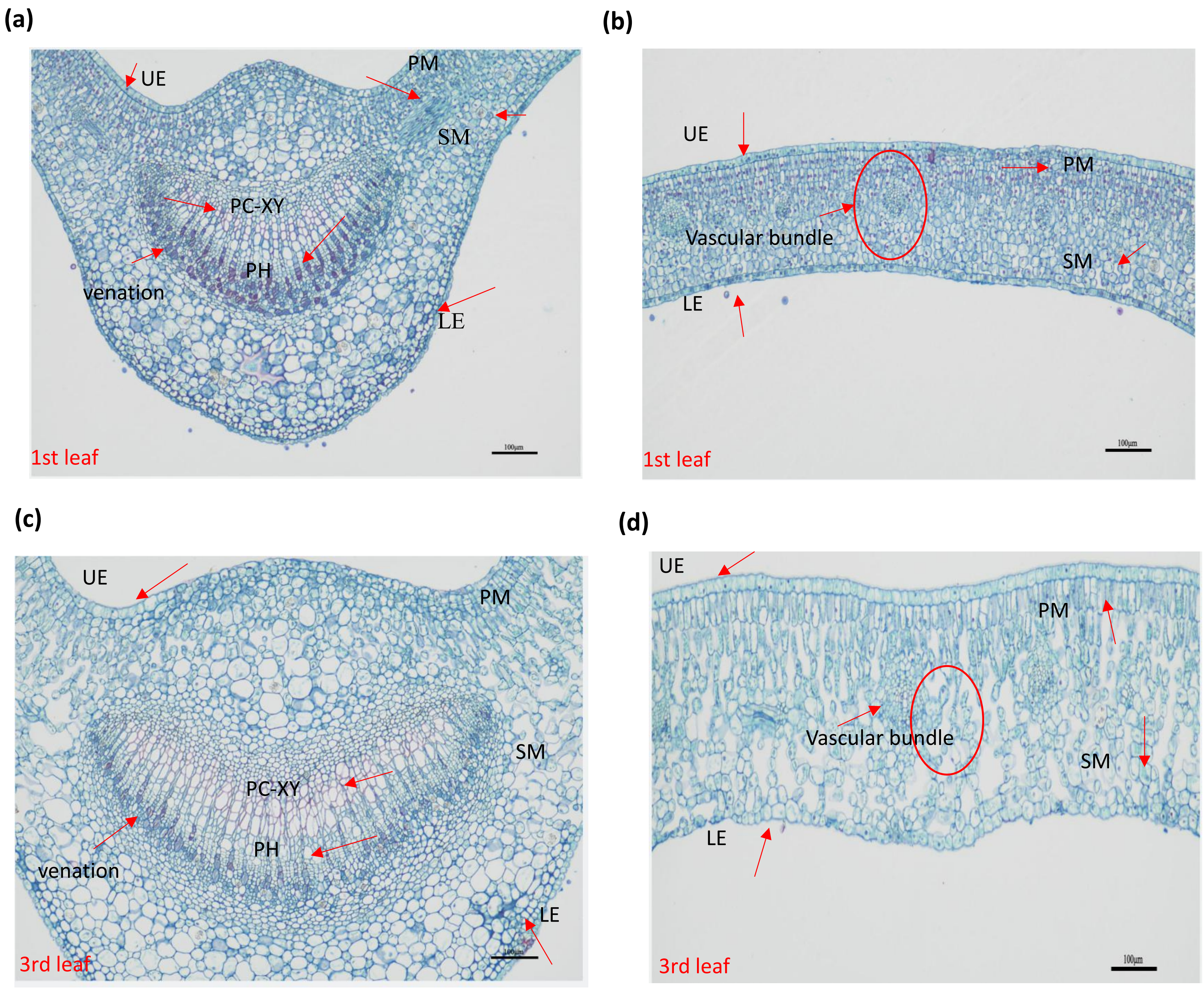

Figure S2

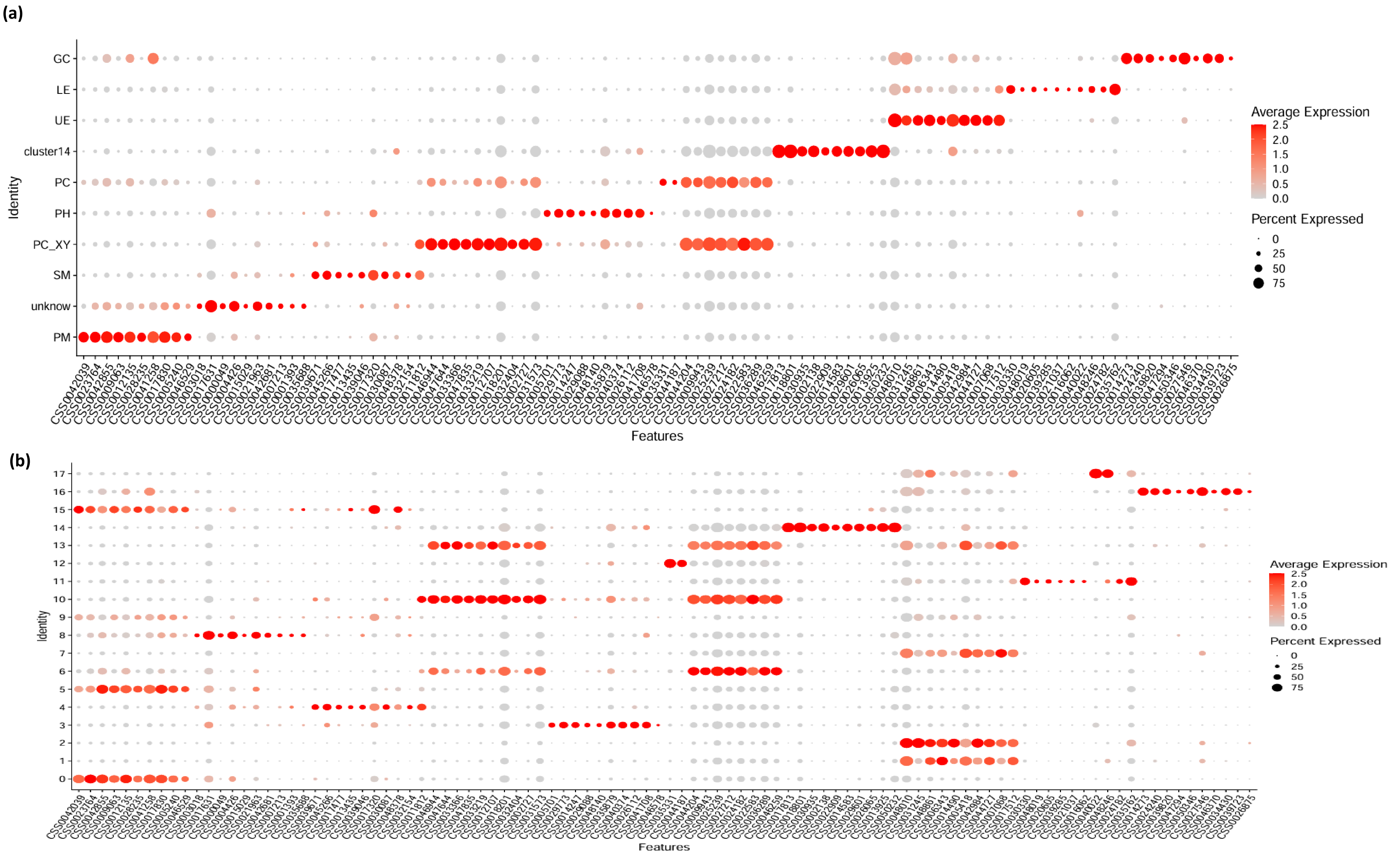
(a)
GC
LE
UE
cluster14
PC
PH
PC_XY
SM
unknow
PM
Identity
Features
Average Expression
2.5
2.0
1.5
1.0
0.5
0.0
Percent Expressed
0
25
50
75
(b)
17
16
15
14
13
12
11
10
9
8
7
6
5
4
3
2
1
0
Identity
Features
Average Expression
2.5
2.0
1.5
1.0
0.5
0.0
Percent Expressed
0
25
50
75

Figure S3

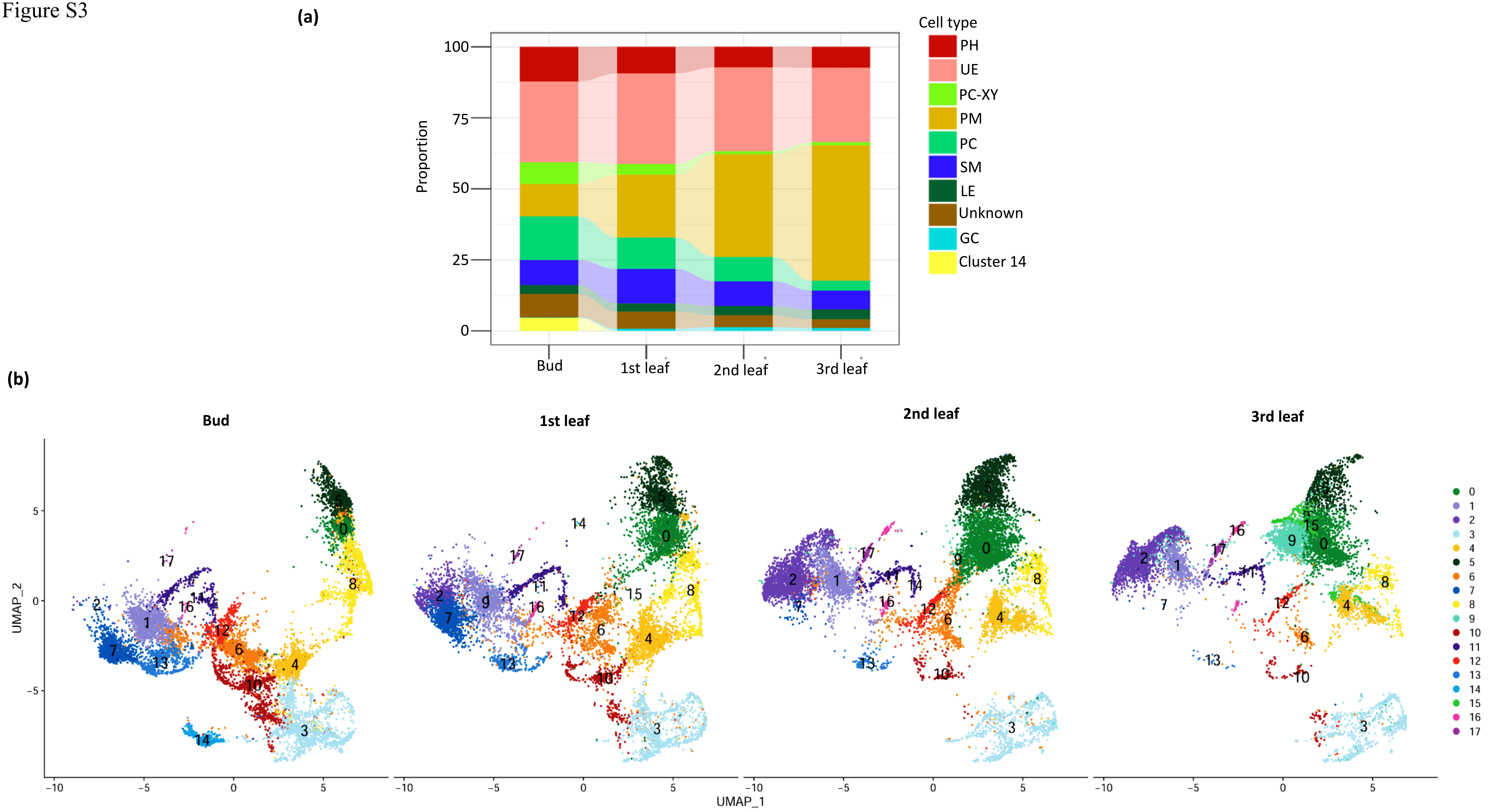

(a)
Cell type
PH
UE
PC-XY
PM
PC
SM
LE
Unknown
GC
Cluster 14
Proportion
100
75
50
25
0
Bud
1st leaf
2nd leaf
3rd leaf
(b)
Bud
1st leaf
2nd leaf
3rd leaf
UMAP_2
UMAP_1

Figure S4

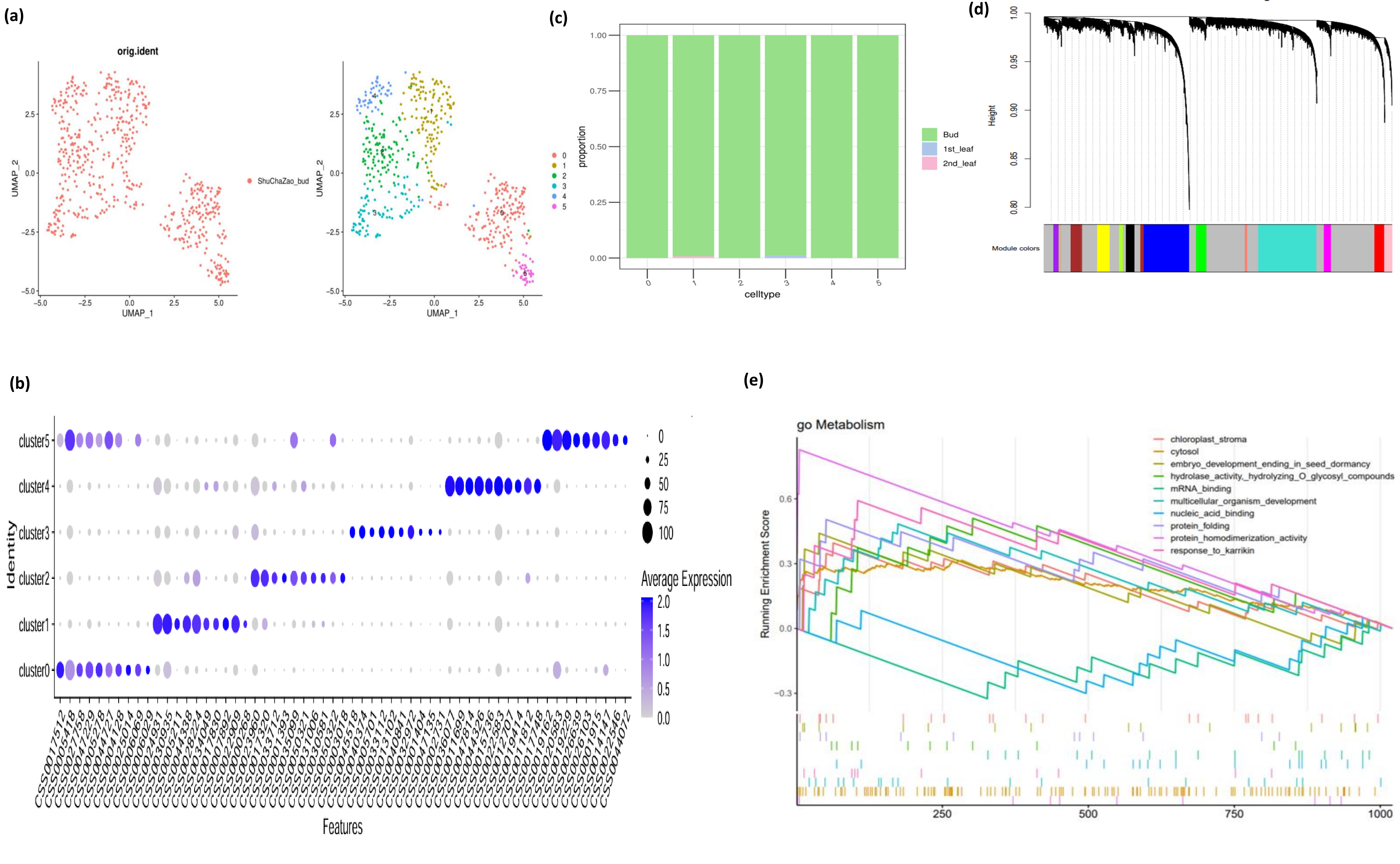
(a)
orig.ident
ShuChaZao_bud
UMAP_1
UMAP_2
(c)
proportion
celltype
Bud
1st_leaf
2nd_leaf
(d)
bud hdWGCNA Dendrogram
Height
Module colors
(b)
cluster5
cluster4
cluster3
cluster2
cluster1
cluster0
Identity
Features
Average Expression
(e)
go Metabolism
Running Enrichment Score
chloroplast_stroma
cytosol
embryo_development_ending_in_seed_dormancy
hydrolase_activity,_hydrolyzing_O_glycosyl_compounds
mRNA_binding
multicellular_organism_development
nucleic_acid_binding
protein_folding
protein_homodimerization_activity
response_to_karrikin

Figure S5

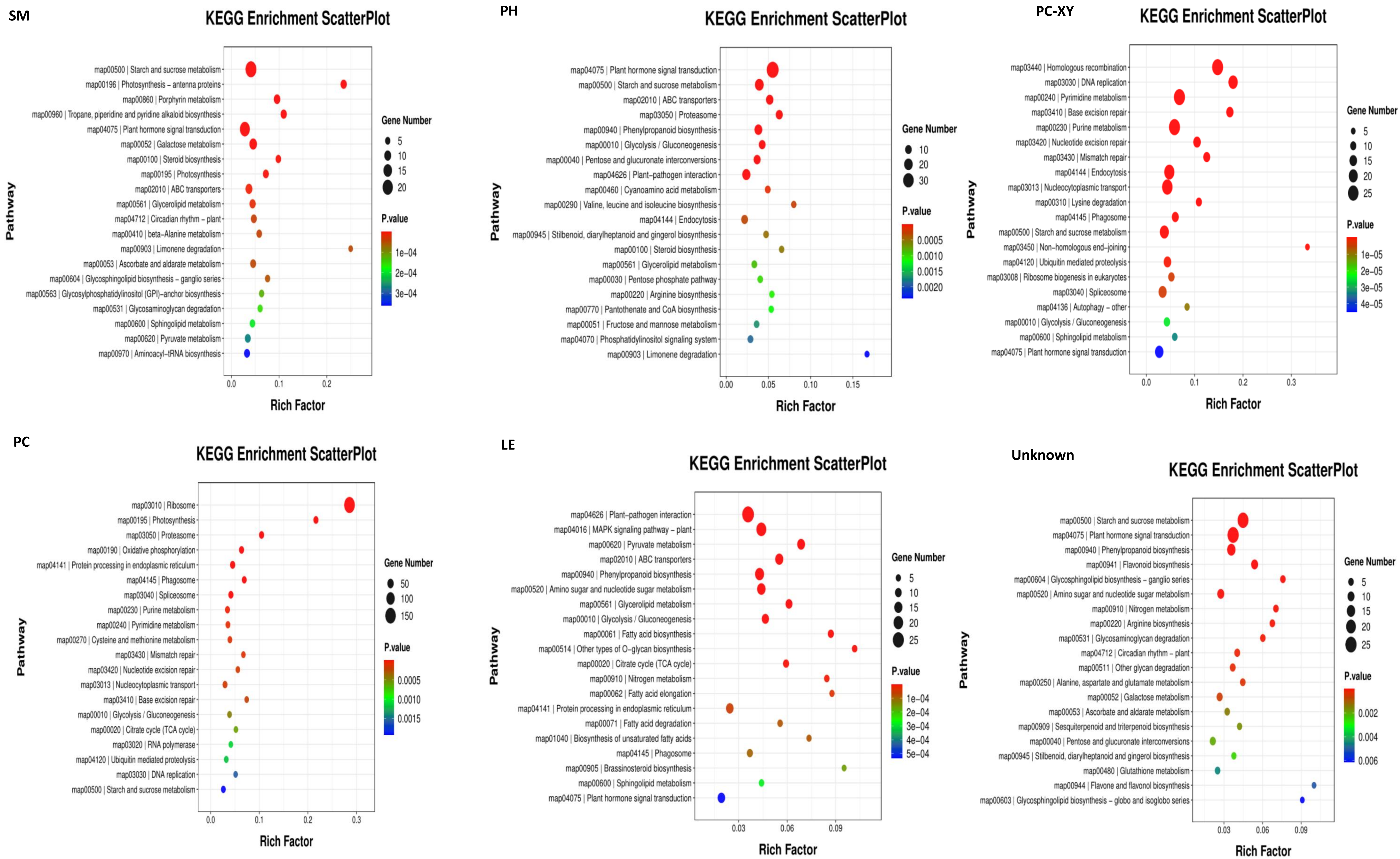
SM
KEGG Enrichment ScatterPlot
map00500 | Starch and sucrose metabolism
map00196 | Photosynthesis – antenna proteins
map00860 | Porphyrin metabolism
map00960 | Tropane, piperidine and pyridine alkaloid biosynthesis
map04075 | Plant hormone signal transduction
map00052 | Galactose metabolism
map00100 | Steroid biosynthesis
map00195 | Photosynthesis
map02010 | ABC transporters
map00561 | Glycerolipid metabolism
map04712 | Circadian rhythm – plant
map00410 | beta–Alanine metabolism
map00903 | Limonene degradation
map00053 | Ascorbate and aldarate metabolism
map00604 | Glycosphingolipid biosynthesis – ganglio series
map00563 | Glycosylphosphatidylinositol (GPI)–anchor biosynthesis
map00531 | Glycosaminoglycan degradation
map00600 | Sphingolipid metabolism
map00620 | Pyruvate metabolism
map00970 | Aminoacyl–tRNA biosynthesis
Pathway
Rich Factor
0.0
0.1
0.2
Gene Number
5
10
15
20
P.value
1e–04
2e–04
3e–04
PH
KEGG Enrichment ScatterPlot
map04075 | Plant hormone signal transduction
map00500 | Starch and sucrose metabolism
map02010 | ABC transporters
map03050 | Proteasome
map00940 | Phenylpropanoid biosynthesis
map00010 | Glycolysis / Gluconeogenesis
map00040 | Pentose and glucuronate interconversions
map04626 | Plant–pathogen interaction
map00460 | Cyanoamino acid metabolism
map00290 | Valine, leucine and isoleucine biosynthesis
map04144 | Endocytosis
map00945 | Stilbenoid, diarylheptanoid and gingerol biosynthesis
map00100 | Steroid biosynthesis
map00561 | Glycerolipid metabolism
map00030 | Pentose phosphate pathway
map00220 | Arginine biosynthesis
map00770 | Pantothenate and CoA biosynthesis
map00051 | Fructose and mannose metabolism
map04070 | Phosphatidylinositol signaling system
map00903 | Limonene degradation
Pathway
Rich Factor
0.00
0.05
0.10
0.15
Gene Number
10
20
30
P.value
0.0005
0.0010
0.0015
0.0020
PC-XY
KEGG Enrichment ScatterPlot
map03440 | Homologous recombination
map03030 | DNA replication
map00240 | Pyrimidine metabolism
map03410 | Base excision repair
map00230 | Purine metabolism
map03420 | Nucleotide excision repair
map03430 | Mismatch repair
map04144 | Endocytosis
map03013 | Nucleocytoplasmic transport
map00310 | Lysine degradation
map04145 | Phagosome
map00500 | Starch and sucrose metabolism
map03450 | Non–homologous end–joining
map04120 | Ubiquitin mediated proteolysis
map03008 | Ribosome biogenesis in eukaryotes
map03040 | Spliceosome
map04136 | Autophagy – other
map00010 | Glycolysis / Gluconeogenesis
map00600 | Sphingolipid metabolism
map04075 | Plant hormone signal transduction
Pathway
Rich Factor
0.0
0.1
0.2
0.3
Gene Number
5
10
15
20
25
P.value
1e–05
2e–05
3e–05
4e–05
PC
KEGG Enrichment ScatterPlot
map03010 | Ribosome
map00195 | Photosynthesis
map03050 | Proteasome
map00190 | Oxidative phosphorylation
map04141 | Protein processing in endoplasmic reticulum
map04145 | Phagosome
map03040 | Spliceosome
map00230 | Purine metabolism
map00240 | Pyrimidine metabolism
map00270 | Cysteine and methionine metabolism
map03430 | Mismatch repair
map03420 | Nucleotide excision repair
map03013 | Nucleocytoplasmic transport
map03410 | Base excision repair
map00010 | Glycolysis / Gluconeogenesis
map00020 | Citrate cycle (TCA cycle)
map03020 | RNA polymerase
map04120 | Ubiquitin mediated proteolysis
map03030 | DNA replication
map00500 | Starch and sucrose metabolism
Pathway
Rich Factor
0.0
0.1
0.2
0.3
Gene Number
50
100
150
P.value
0.0005
0.0010
0.0015
LE
KEGG Enrichment ScatterPlot
map04626 | Plant–pathogen interaction
map04016 | MAPK signaling pathway – plant
map00620 | Pyruvate metabolism
map02010 | ABC transporters
map00940 | Phenylpropanoid biosynthesis
map00520 | Amino sugar and nucleotide sugar metabolism
map00561 | Glycerolipid metabolism
map00010 | Glycolysis / Gluconeogenesis
map00061 | Fatty acid biosynthesis
map00514 | Other types of O–glycan biosynthesis
map00020 | Citrate cycle (TCA cycle)
map00910 | Nitrogen metabolism
map00062 | Fatty acid elongation
map04141 | Protein processing in endoplasmic reticulum
map00071 | Fatty acid degradation
map01040 | Biosynthesis of unsaturated fatty acids
map04145 | Phagosome
map00905 | Brassinosteroid biosynthesis
map00600 | Sphingolipid metabolism
map04075 | Plant hormone signal transduction
Pathway
Rich Factor
0.03
0.06
0.09
Gene Number
5
10
15
20
25
P.value
1e–04
2e–04
3e–04
4e–04
5e–04
Unknown
KEGG Enrichment ScatterPlot
map00500 | Starch and sucrose metabolism
map04075 | Plant hormone signal transduction
map00940 | Phenylpropanoid biosynthesis
map00941 | Flavonoid biosynthesis
map00604 | Glycosphingolipid biosynthesis – ganglio series
map00520 | Amino sugar and nucleotide sugar metabolism
map00910 | Nitrogen metabolism
map00220 | Arginine biosynthesis
map00531 | Glycosaminoglycan degradation
map04712 | Circadian rhythm – plant
map00511 | Other glycan degradation
map00250 | Alanine, aspartate and glutamate metabolism
map00052 | Galactose metabolism
map00053 | Ascorbate and aldarate metabolism
map00909 | Sesquiterpenoid and triterpenoid biosynthesis
map00040 | Pentose and glucuronate interconversions
map00945 | Stilbenoid, diarylheptanoid and gingerol biosynthesis
map00480 | Glutathione metabolism
map00944 | Flavone and flavonol biosynthesis
map00603 | Glycosphingolipid biosynthesis – globo and isoglobo series
Pathway
Rich Factor
0.03
0.06
0.09
Gene Number
5
10
15
20
25
P.value
0.002
0.004
0.006

GC

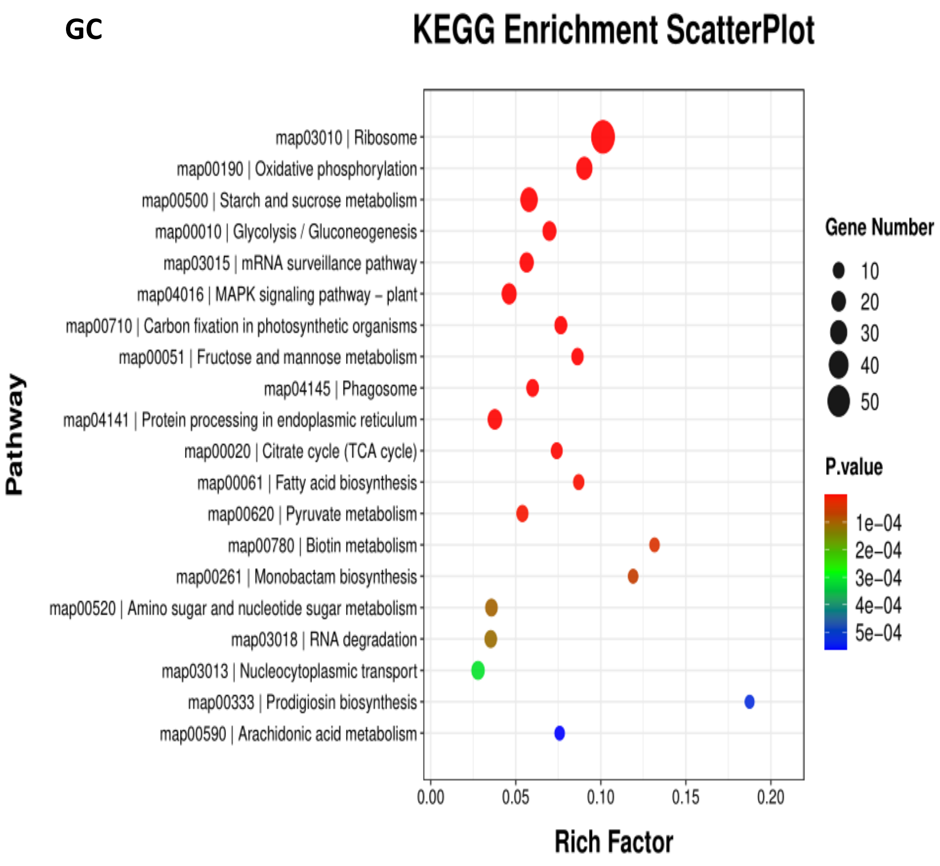

KEGG Enrichment ScatterPlot
map03010 | Ribosome
map00190 | Oxidative phosphorylation
map00500 | Starch and sucrose metabolism
map00010 | Glycolysis / Gluconeogenesis
map03015 | mRNA surveillance pathway
map04016 | MAPK signaling pathway – plant
map00710 | Carbon fixation in photosynthetic organisms
map00051 | Fructose and mannose metabolism
map04145 | Phagosome
map04141 | Protein processing in endoplasmic reticulum
map00020 | Citrate cycle (TCA cycle)
map00061 | Fatty acid biosynthesis
map00620 | Pyruvate metabolism
map00780 | Biotin metabolism
map00261 | Monobactam biosynthesis
map00520 | Amino sugar and nucleotide sugar metabolism
map03018 | RNA degradation
map03013 | Nucleocytoplasmic transport
map00333 | Prodigiosin biosynthesis
map00590 | Arachidonic acid metabolism
Pathway
0.00
0.05
0.10
0.15
0.20
Rich Factor
Gene Number
10
20
30
40
50
P.value
1e−04
2e−04
3e−04
4e−04
5e−04

UE

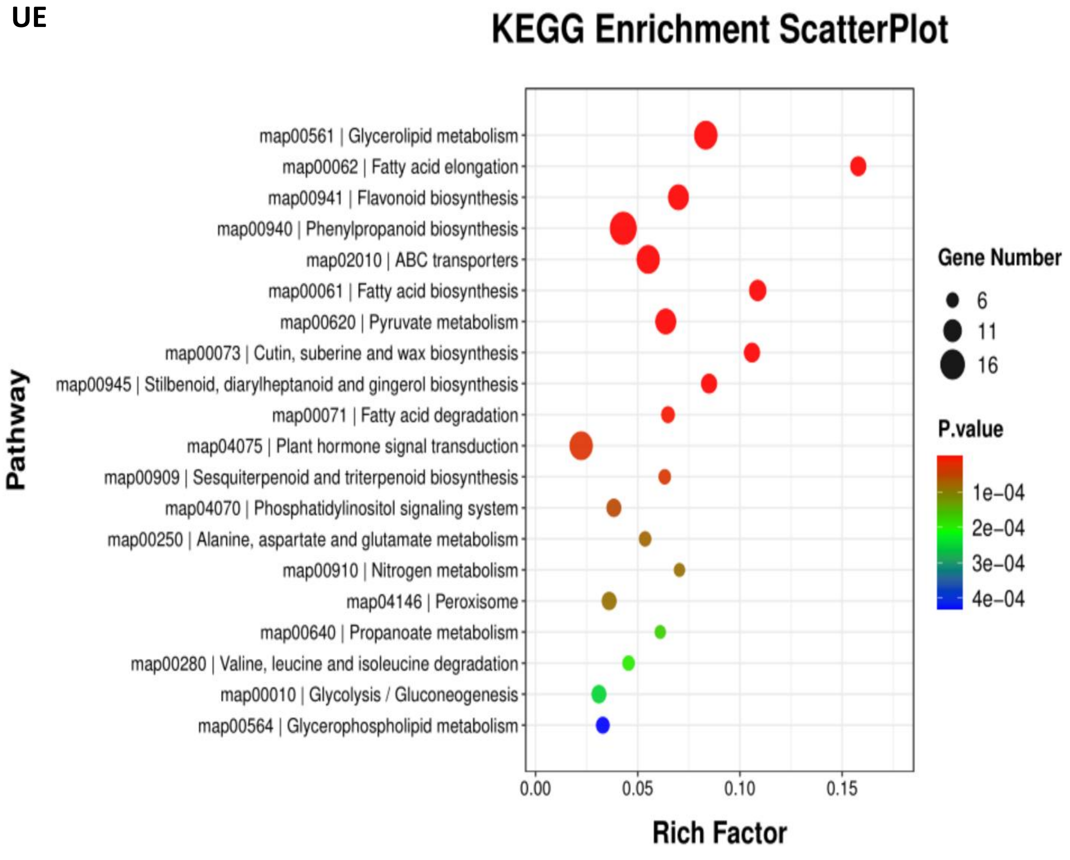

KEGG Enrichment ScatterPlot
map00561 | Glycerolipid metabolism
map00062 | Fatty acid elongation
map00941 | Flavonoid biosynthesis
map00940 | Phenylpropanoid biosynthesis
map02010 | ABC transporters
map00061 | Fatty acid biosynthesis
map00620 | Pyruvate metabolism
map00073 | Cutin, suberine and wax biosynthesis
map00945 | Stilbenoid, diarylheptanoid and gingerol biosynthesis
map00071 | Fatty acid degradation
map04075 | Plant hormone signal transduction
map00909 | Sesquiterpenoid and triterpenoid biosynthesis
map04070 | Phosphatidylinositol signaling system
map00250 | Alanine, aspartate and glutamate metabolism
map00910 | Nitrogen metabolism
map04146 | Peroxisome
map00640 | Propanoate metabolism
map00280 | Valine, leucine and isoleucine degradation
map00010 | Glycolysis / Gluconeogenesis
map00564 | Glycerophospholipid metabolism
Pathway
0.00
0.05
0.10
0.15
Rich Factor
Gene Number
6
11
16
P.value
1e−04
2e−04
3e−04
4e−04

Figure S6

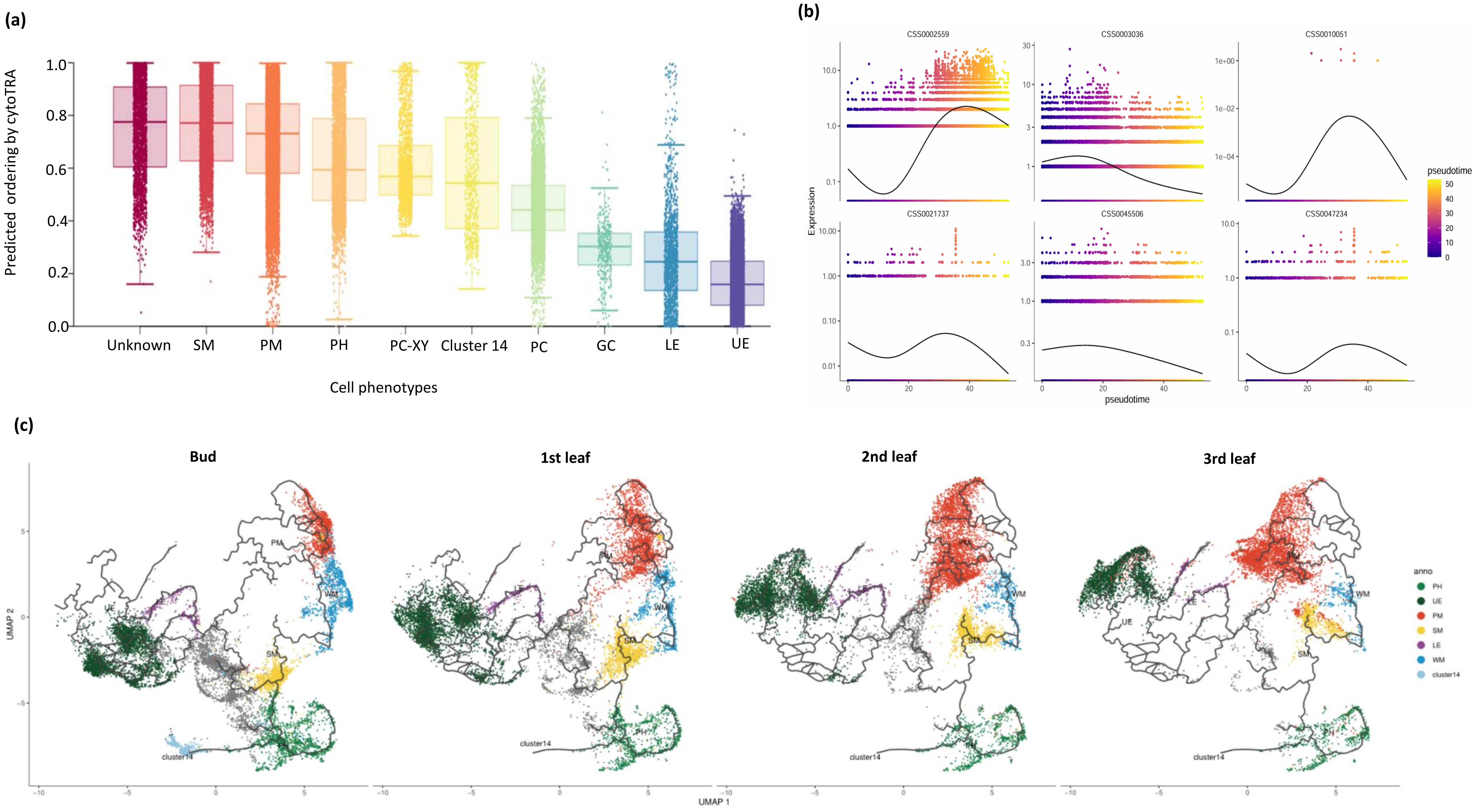

(a)
Predicted ordering by cytoTRA
1.0
0.8
0.6
0.4
0.2
0.0
Unknown
SM
PM
PH
PC-XY
Cluster 14
PC
GC
LE
UE
Cell phenotypes
(b)
CSS0002559
CSS0003036
CSS0010051
CSS0021737
CSS0045506
CSS0047234
Expression
pseudotime
(c)
Bud
1st leaf
2nd leaf
3rd leaf
UMAP 2
UMAP 1
anno
PH
UE
PM
SM
LE
WM
cluster14

Figure S7

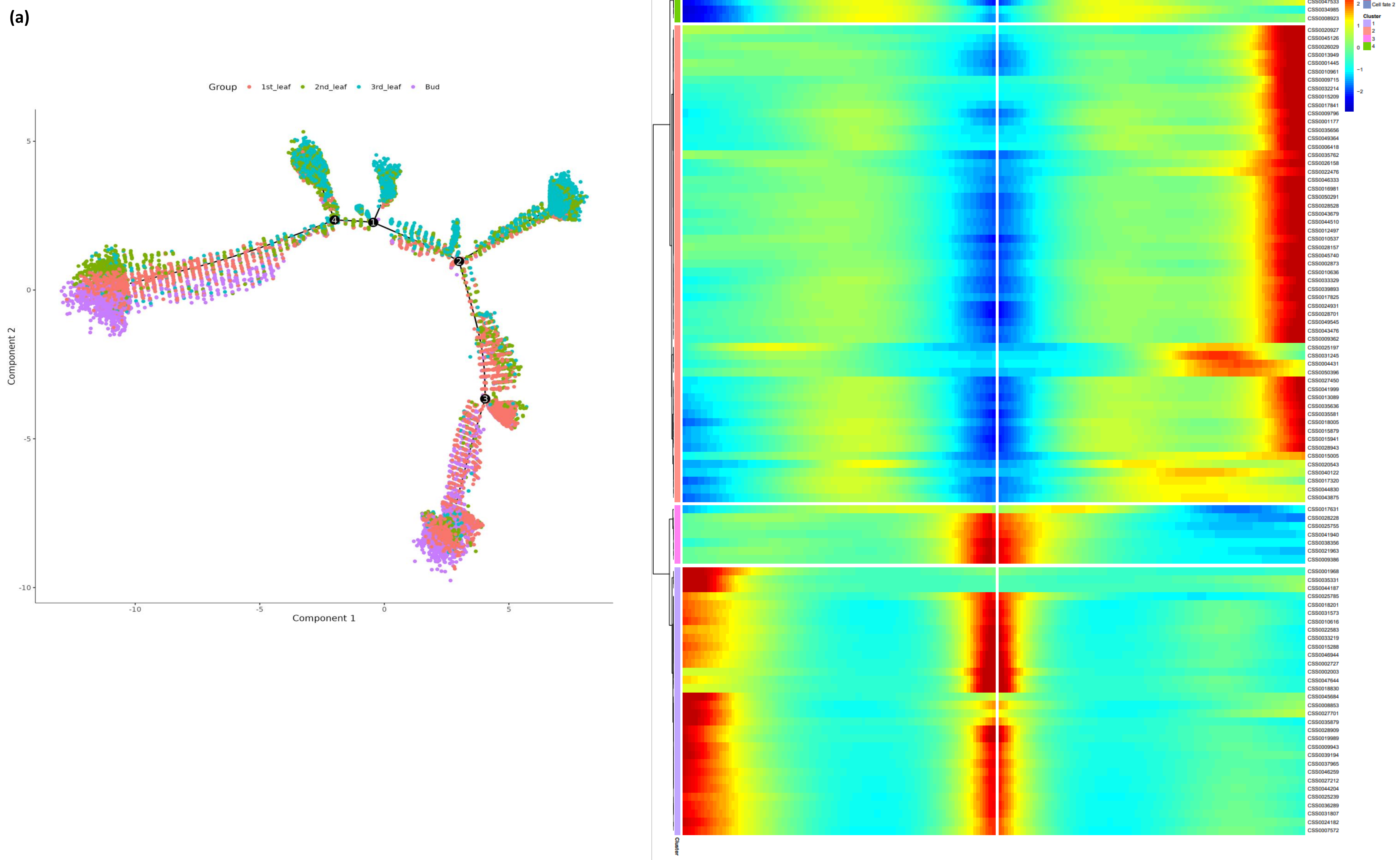
(a)
Group
1st_leaf
2nd_leaf
3rd_leaf
Bud
5
0
-5
-10
Component 2
-10
-5
0
5
Component 1
1
2
3
4
(b)
Cell Type
Pre-branch
Cell fate 1
Cell fate 2
Cluster
1
2
3
4
3
2
1
0
-1
-2
Cluster

Figure S8

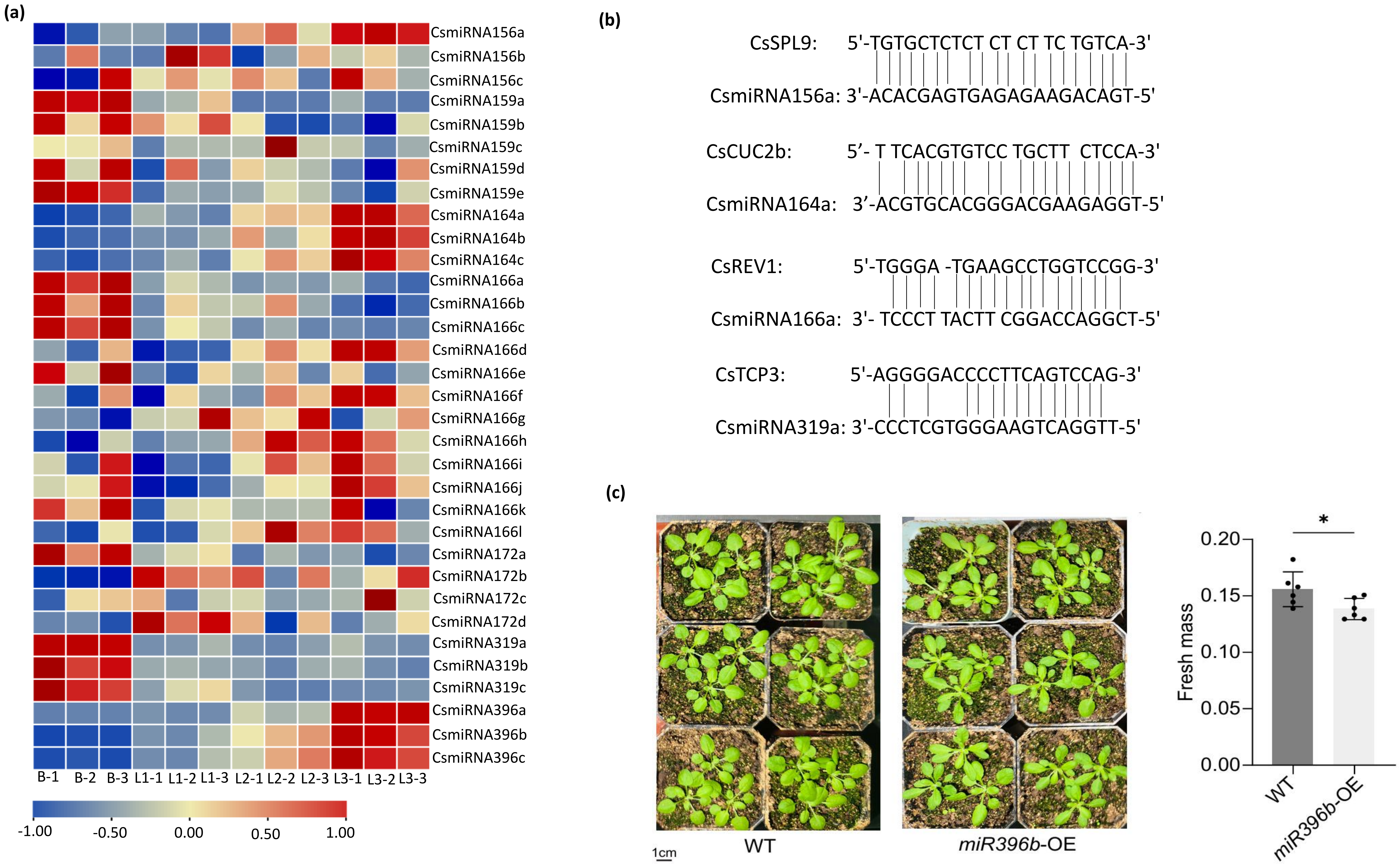
(a)
CsmiRNA156a
CsmiRNA156b
CsmiRNA156c
CsmiRNA159a
CsmiRNA159b
CsmiRNA159c
CsmiRNA159d
CsmiRNA159e
CsmiRNA164a
CsmiRNA164b
CsmiRNA164c
CsmiRNA166a
CsmiRNA166b
CsmiRNA166c
CsmiRNA166d
CsmiRNA166e
CsmiRNA166f
CsmiRNA166g
CsmiRNA166h
CsmiRNA166i
CsmiRNA166j
CsmiRNA166k
CsmiRNA166l
CsmiRNA172a
CsmiRNA172b
CsmiRNA172c
CsmiRNA172d
CsmiRNA319a
CsmiRNA319b
CsmiRNA319c
CsmiRNA396a
CsmiRNA396b
CsmiRNA396c
B-1 B-2 B-3 L1-1 L1-2 L1-3 L2-1 L2-2 L2-3 L3-1 L3-2 L3-3
-1.00 -0.50 0.00 0.50 1.00
(b)
CsSPL9: 5'-TGTGCTCTCT CT CT TC TGTCA-3'
CsmiRNA156a: 3'-ACACGAGTGAGAGAAGACAGT-5'
CsCUC2b: 5'- T TCACGTGTCC TGCTT CTCCA-3'
CsmiRNA164a: 3'-ACGTGCACGGGACGAAGAGGT-5'
CsREV1: 5'-TGGGA -TGAAGCCTGGTCCGG-3'
CsmiRNA166a: 3'- TCCCT TACTT CGGACCAGGCT-5'
CsTCP3: 5'-AGGGGACCCCTTCAGTCCAG-3'
CsmiRNA319a: 3'-CCCTCGTGGGAAGTCAGGTT-5'
(c)
1cm
WT
miR396b-OE
*
0.20
0.15
0.10
0.05
0.00
Fresh mass
WT
miR396b-OE

Figure S9

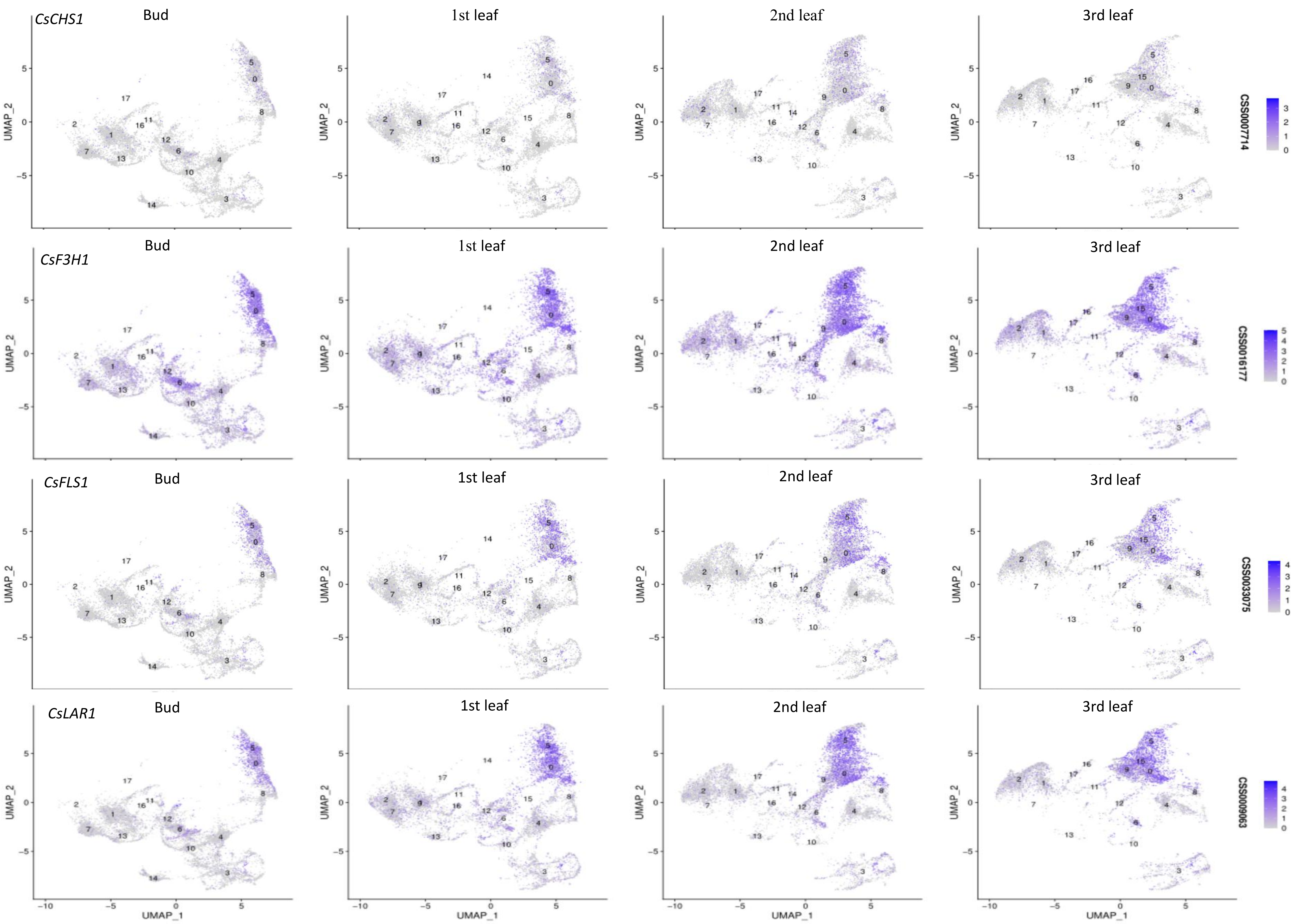
CsCHS1
Bud
1st leaf
2nd leaf
3rd leaf
CSS0007714
CsF3H1
CSS0016177
CsFLS1
CSS0033075
CsLAR1
CSS0009063
UMAP_1
UMAP_2

Figure S10

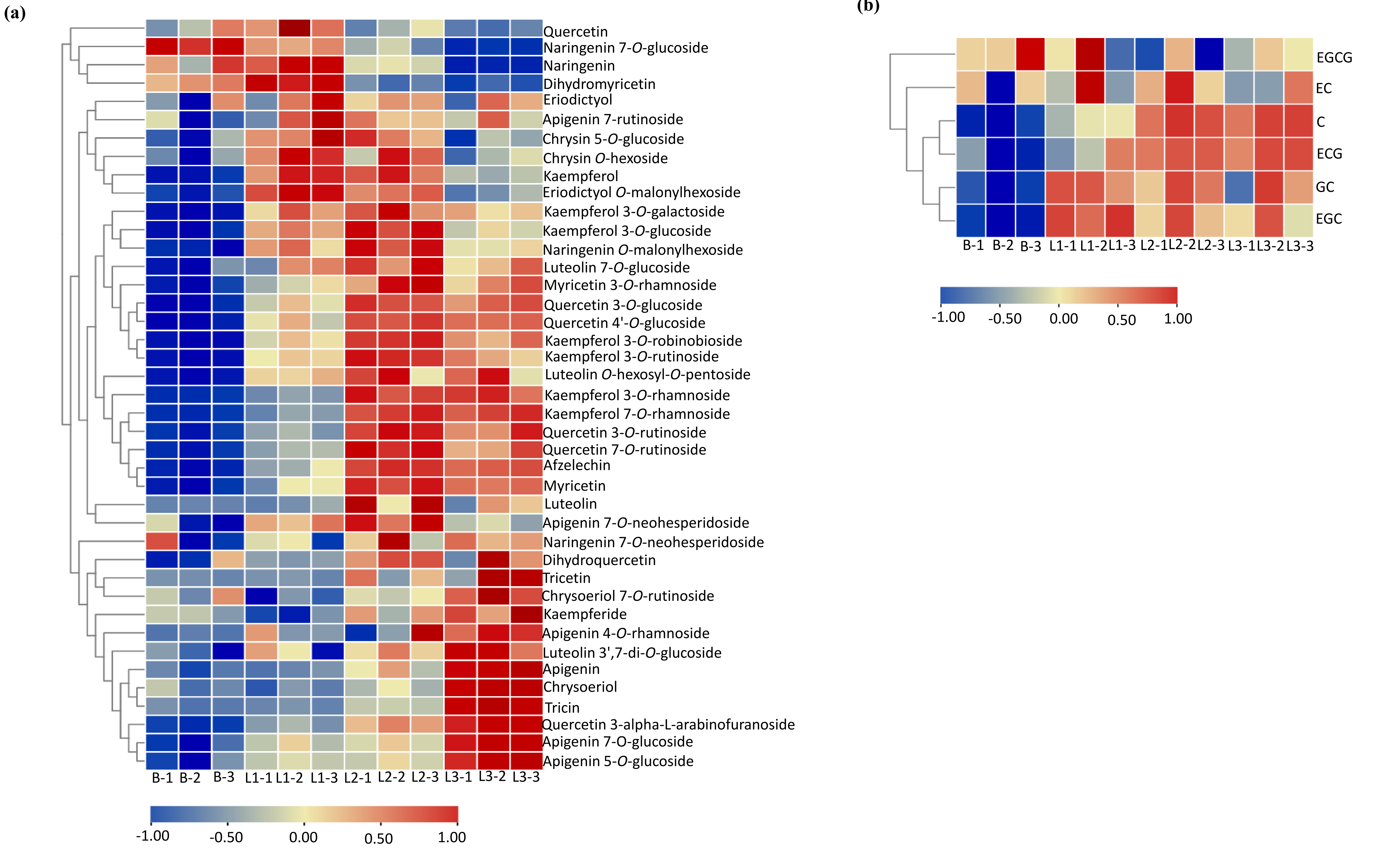
(a)
Quercetin
Naringenin 7-O-glucoside
Naringenin
Dihydromyricetin
Eriodictyol
Apigenin 7-rutinoside
Chrysin 5-O-glucoside
Chrysin O-hexoside
Kaempferol
Eriodictyol O-malonylhexoside
Kaempferol 3-O-galactoside
Kaempferol 3-O-glucoside
Naringenin O-malonylhexoside
Luteolin 7-O-glucoside
Myricetin 3-O-rhamnoside
Quercetin 3-O-glucoside
Quercetin 4'-O-glucoside
Kaempferol 3-O-robinobioside
Kaempferol 3-O-rutinoside
Luteolin O-hexosyl-O-pentoside
Kaempferol 3-O-rhamnoside
Kaempferol 7-O-rhamnoside
Quercetin 3-O-rutinoside
Quercetin 7-O-rutinoside
Afzelechin
Myricetin
Luteolin
Apigenin 7-O-neohesperidoside
Naringenin 7-O-neohesperidoside
Dihydroquercetin
Tricetin
Chrysoeriol 7-O-rutinoside
Kaempferide
Apigenin 4-O-rhamnoside
Luteolin 3',7-di-O-glucoside
Apigenin
Chrysoeriol
Tricin
Quercetin 3-alpha-L-arabinofuranoside
Apigenin 7-O-glucoside
Apigenin 5-O-glucoside
B-1 B-2 B-3 L1-1 L1-2 L1-3 L2-1 L2-2 L2-3 L3-1 L3-2 L3-3
-1.00 -0.50 0.00 0.50 1.00
(b)
EGCG
EC
C
ECG
GC
EGC
B-1 B-2 B-3 L1-1 L1-2 L1-3 L2-1 L2-2 L2-3 L3-1 L3-2 L3-3
-1.00 -0.50 0.00 0.50 1.00

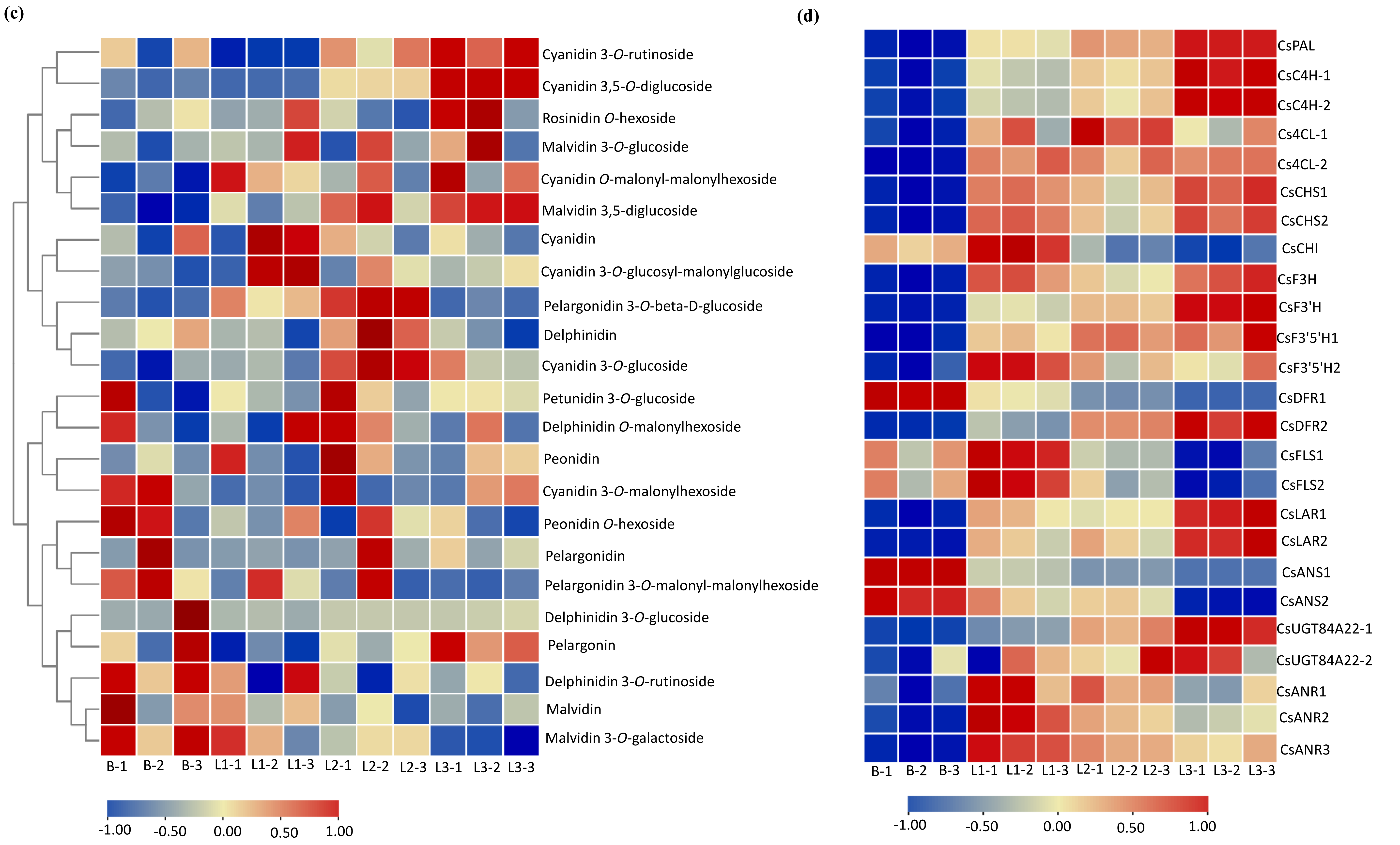
(c)
Cyanidin 3-O-rutinoside
Cyanidin 3,5-O-diglucoside
Rosinidin O-hexoside
Malvidin 3-O-glucoside
Cyanidin O-malonyl-malonylhexoside
Malvidin 3,5-diglucoside
Cyanidin
Cyanidin 3-O-glucosyl-malonylglucoside
Pelargonidin 3-O-beta-D-glucoside
Delphinidin
Cyanidin 3-O-glucoside
Petunidin 3-O-glucoside
Delphinidin O-malonylhexoside
Peonidin
Cyanidin 3-O-malonylhexoside
Peonidin O-hexoside
Pelargonidin
Pelargonidin 3-O-malonyl-malonylhexoside
Delphinidin 3-O-glucoside
Pelargonin
Delphinidin 3-O-rutinoside
Malvidin
Malvidin 3-O-galactoside
B-1 B-2 B-3 L1-1 L1-2 L1-3 L2-1 L2-2 L2-3 L3-1 L3-2 L3-3
-1.00 -0.50 0.00 0.50 1.00
(d)
CsPAL
CsC4H-1
CsC4H-2
Cs4CL-1
Cs4CL-2
CsCHS1
CsCHS2
CsCHI
CsF3H
CsF3'H
CsF3'5'H1
CsF3'5'H2
CsDFR1
CsDFR2
CsFLS1
CsFLS2
CsLAR1
CsLAR2
CsANS1
CsANS2
CsUGT84A22-1
CsUGT84A22-2
CsANR1
CsANR2
CsANR3
B-1 B-2 B-3 L1-1 L1-2 L1-3 L2-1 L2-2 L2-3 L3-1 L3-2 L3-3
-1.00 -0.50 0.00 0.50 1.00

Figure S11

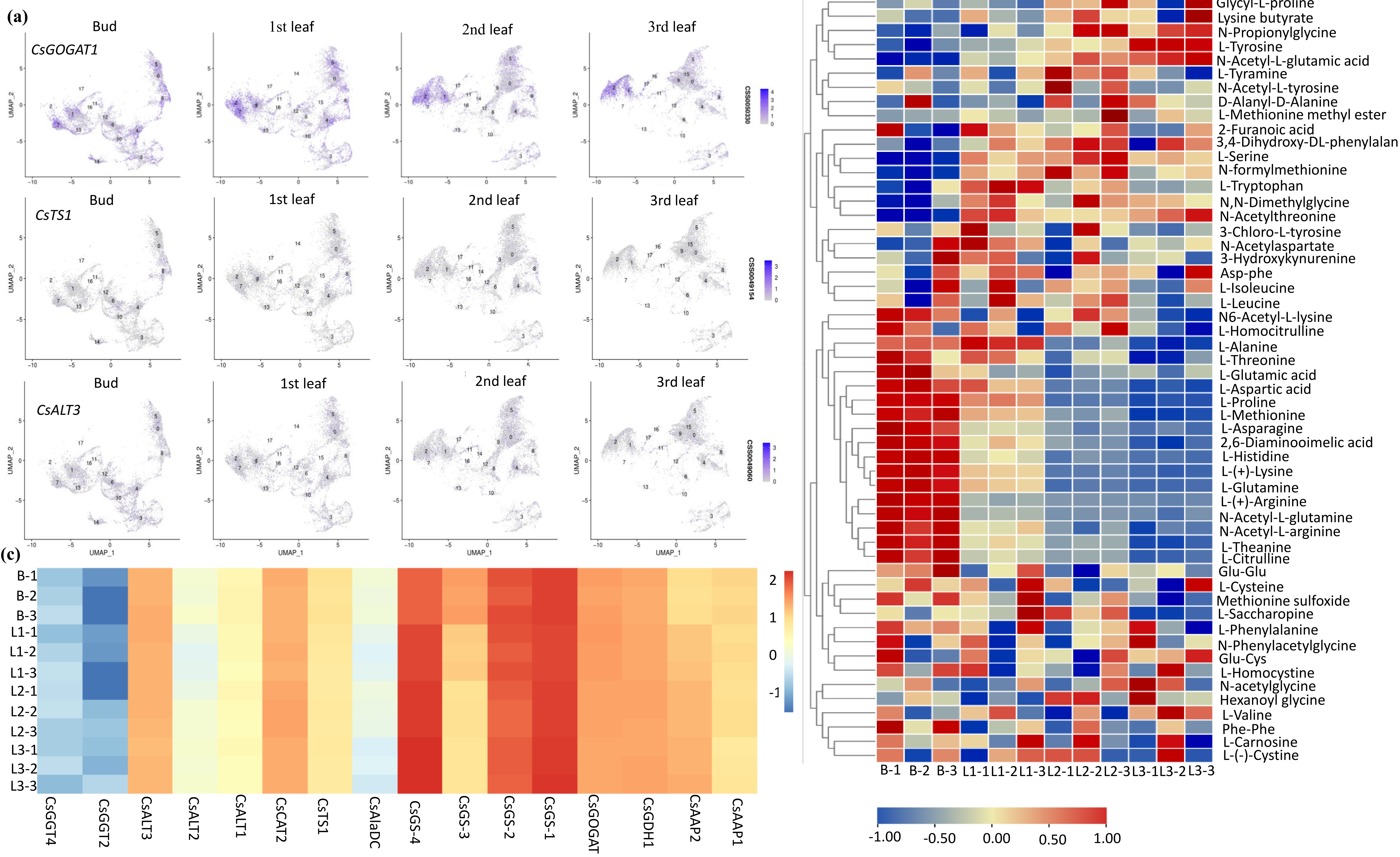
(a)
Bud
1st leaf
2nd leaf
3rd leaf
CsGOGAT1
CsTS1
CsALT3
UMAP_1
UMAP_2
CSS0050330
CSS0049154
CSS0049060
(b)
Glycyl-L-proline
Lysine butyrate
N-Propionylglycine
L-Tyrosine
N-Acetyl-L-glutamic acid
L-Tyramine
N-Acetyl-L-tyrosine
D-Alanyl-D-Alanine
L-Methionine methyl ester
2-Furanoic acid
3,4-Dihydroxy-DL-phenylalan
L-Serine
N-formylmethionine
L-Tryptophan
N,N-Dimethylglycine
N-Acetylthreonine
3-Chloro-L-tyrosine
N-Acetylaspartate
3-Hydroxykynurenine
Asp-phe
L-Isoleucine
L-Leucine
N6-Acetyl-L-lysine
L-Homocitrulline
L-Alanine
L-Threonine
L-Glutamic acid
L-Aspartic acid
L-Proline
L-Methionine
L-Asparagine
2,6-Diaminooimelic acid
L-Histidine
L-(+)-Lysine
L-Glutamine
L-(+)-Arginine
N-Acetyl-L-glutamine
N-Acetyl-L-arginine
L-Theanine
L-Citrulline
Glu-Glu
L-Cysteine
Methionine sulfoxide
L-Saccharopine
L-Phenylalanine
N-Phenylacetylglycine
Glu-Cys
L-Homocystine
N-acetylglycine
Hexanoyl glycine
L-Valine
Phe-Phe
L-Carnosine
L-(-)-Cystine
B-1 B-2 B-3 L1-1 L1-2 L1-3 L2-1 L2-2 L2-3 L3-1 L3-2 L3-3
-1.00 -0.50 0.00 0.50 1.00
(c)
B-1 B-2 B-3 L1-1 L1-2 L1-3 L2-1 L2-2 L2-3 L3-1 L3-2 L3-3
2 1 0 -1
CsGGT4 CsGGT2 CsALT3 CsALT2 CsALT1 CsCAT2 CsTS1 CsAlaDC CsGS-4 CsGS-3 CsGS-2 CsGS-1 CsGOGAT CsGDH1 CsAAP2 CsAAP1

Figure S12

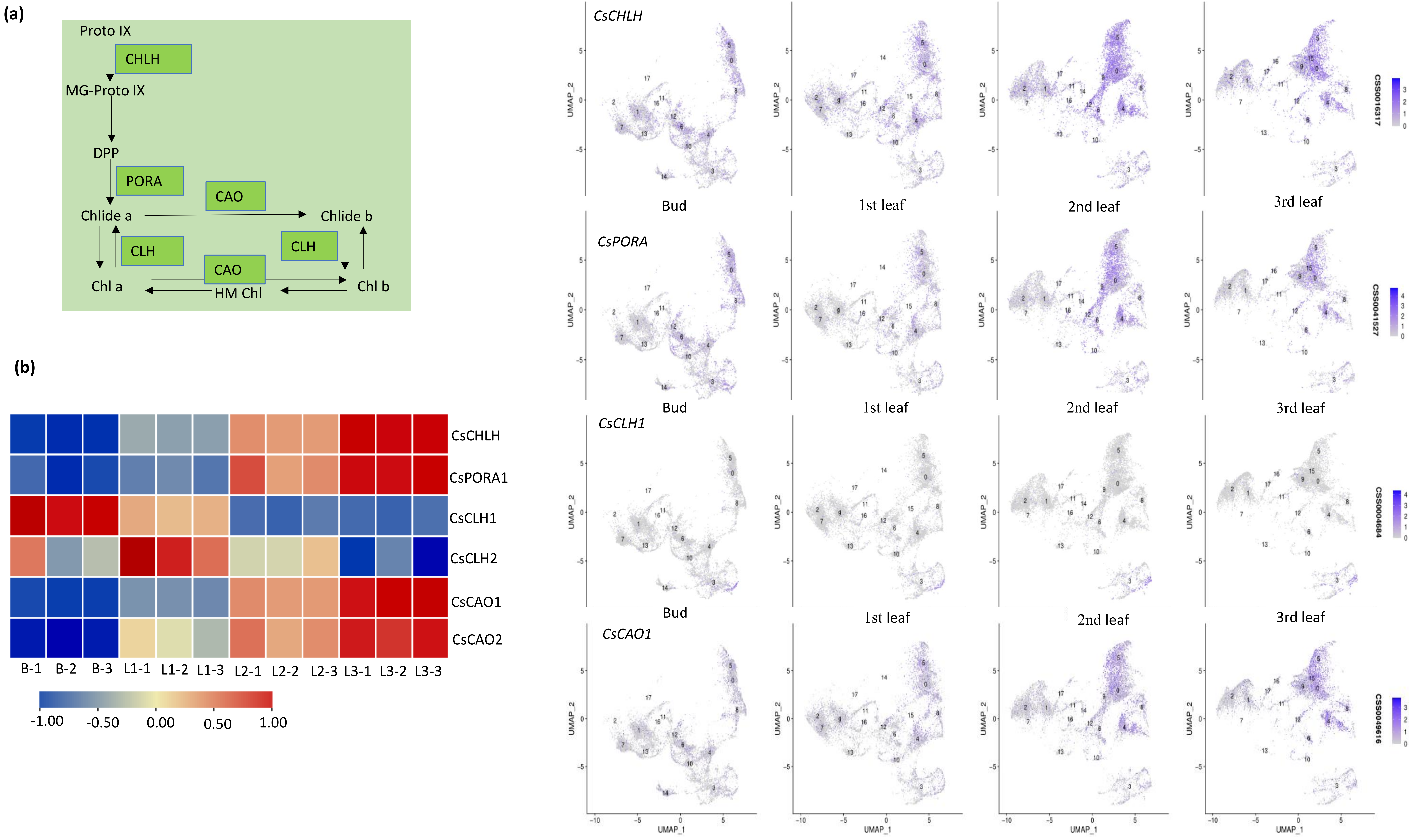
(a)
Proto IX
CHLH
MG-Proto IX
DPP
PORA
CAO
Chlide a
Chlide b
CLH
CLH
CAO
Chl a
HM Chl
Chl b
(b)
CsCHLH
CsPORA1
CsCLH1
CsCLH2
CsCAO1
CsCAO2
B-1 B-2 B-3 L1-1 L1-2 L1-3 L2-1 L2-2 L2-3 L3-1 L3-2 L3-3
-1.00 -0.50 0.00 0.50 1.00
(c)
Bud
1st leaf
2nd leaf
3rd leaf
CsCHLH
CsPORA
CsCLH1
CsCAO1
UMAP_1
UMAP_2

Figure S13

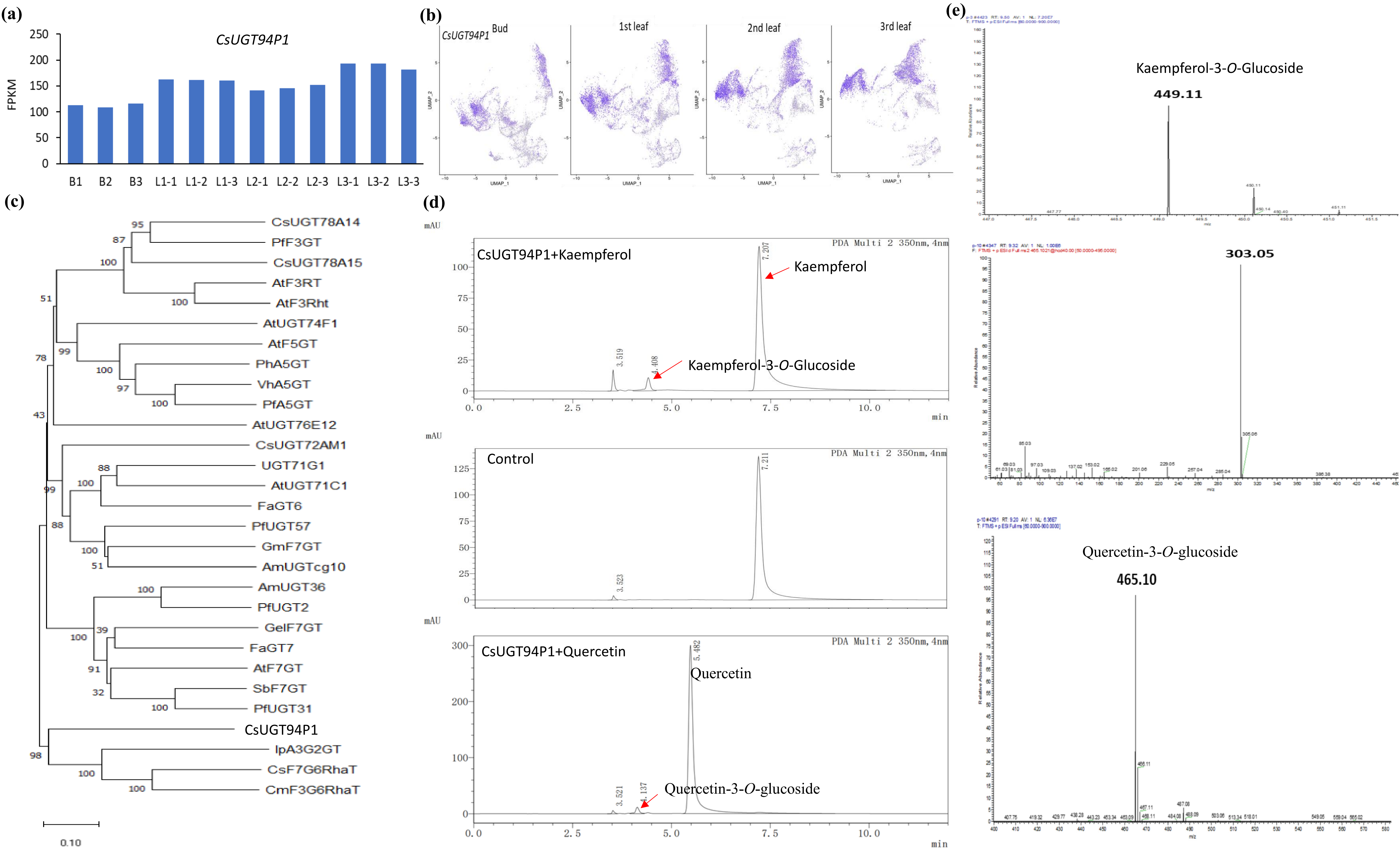

(a)
CsUGT94P1
FPKM
250
200
150
100
50
0
B1
B2
B3
L1-1
L1-2
L1-3
L2-1
L2-2
L2-3
L3-1
L3-2
L3-3
(b)
CsUGT94P1
Bud
1st leaf
2nd leaf
3rd leaf
(c)
CsUGT78A14
PfF3GT
CsUGT78A15
AtF3RT
AtF3Rht
AtUGT74F1
AtF5GT
PhA5GT
VhA5GT
PfA5GT
AtUGT76E12
CsUGT72AM1
UGT71G1
AtUGT71C1
FaGT6
PfUGT57
GmF7GT
AmUGTcg10
AmUGT36
PfUGT2
GelF7GT
FaGT7
AtF7GT
SbF7GT
PfUGT31
CsUGT94P1
IpA3G2GT
CsF7G6RhaT
CmF3G6RhaT
0.10
(d)
CsUGT94P1+Kaempferol
Kaempferol
Kaempferol-3-O-Glucoside
Control
CsUGT94P1+Quercetin
Quercetin
Quercetin-3-O-glucoside
mAU
min
PDA Multi 2 350nm, 4nm
(e)
Kaempferol-3-O-Glucoside
449.11
303.05
Quercetin-3-O-glucoside
465.10

Figure S14

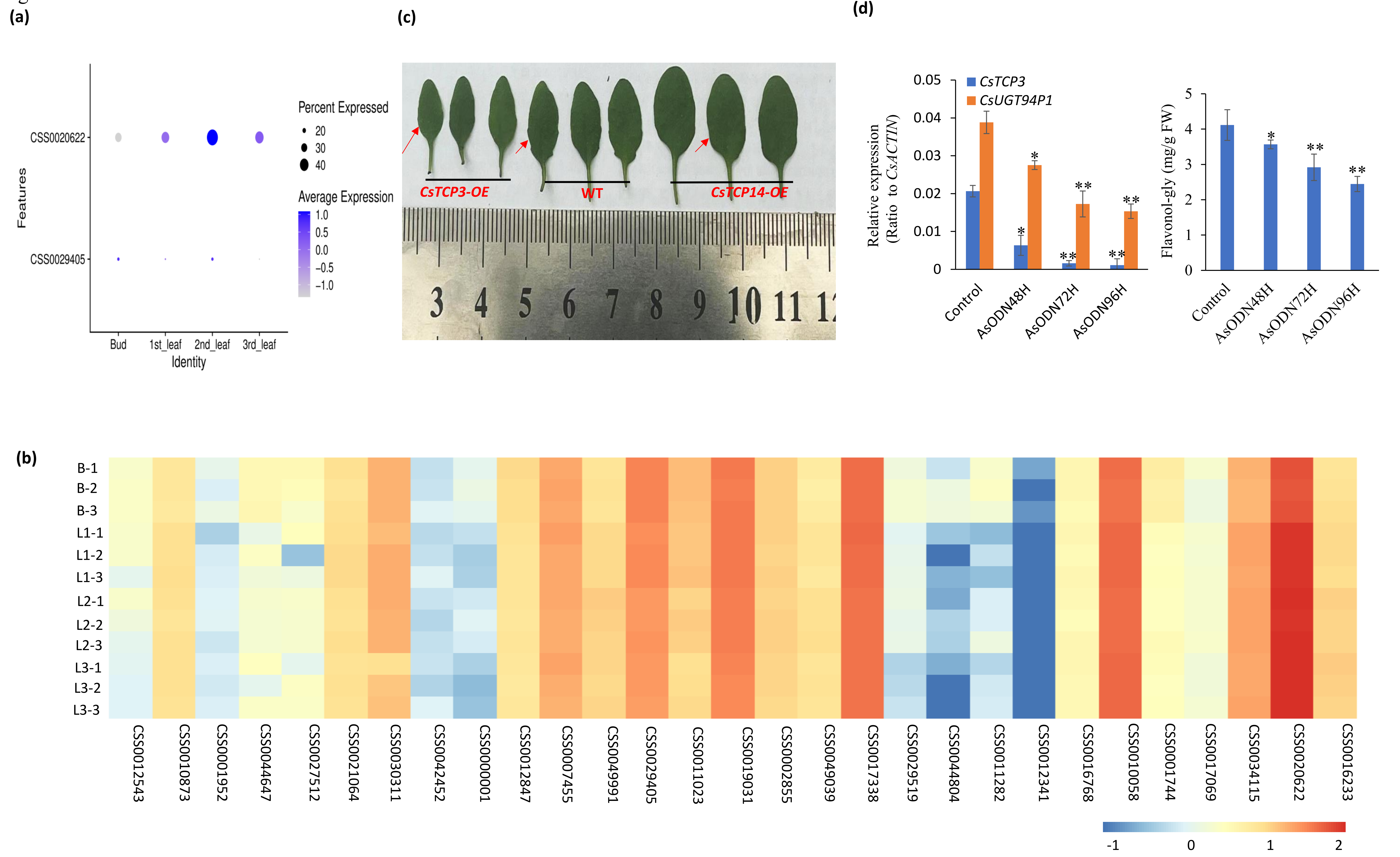

(a)
CSS0020622
CSS0029405
Features
Bud
1st_leaf
2nd_leaf
3rd_leaf
Identity
Percent Expressed
20
30
40
Average Expression
1.0
0.5
0.0
−0.5
−1.0
(c)
CsTCP3-OE
WT
CsTCP14-OE
(d)
CsTCP3
CsUGT94P1
Relative expression (Ratio to CsACTIN)
0.05
0.04
0.03
0.02
0.01
0
Control
AsODN48H
AsODN72H
AsODN96H
Flavonol-gly (mg/g FW)
5
4
3
2
1
0
(b)
B-1
B-2
B-3
L1-1
L1-2
L1-3
L2-1
L2-2
L2-3
L3-1
L3-2
L3-3
CSS0012543
CSS0010873
CSS0001952
CSS0044647
CSS0027512
CSS0021064
CSS0030311
CSS0042452
CSS0000001
CSS0012847
CSS0007455
CSS0049991
CSS0029405
CSS0011023
CSS0019031
CSS0002855
CSS0049039
CSS0017338
CSS0029519
CSS0044804
CSS0011282
CSS0012341
CSS0016768
CSS0010058
CSS0001744
CSS0017069
CSS0034115
CSS0020622
CSS0016233
-1
0
1
2
