# Supplementary material for: Integrated single-nucleus transcriptomic and metabolomic insights into bud-to-leaf development and metabolite synthesis in tea plant
Source: Hortic Res. 2025 Oct 11;13(1):uhaf281. doi: 10.1093/hr/uhaf281 (PMC12871078; doi:10.1093/hr/uhaf281)
Supplement: Web_Material_uhaf281 [file web_material_uhaf281.zip › Supplemental-Methods-S1.docx]

**Integrated single-nucleus transcriptomic and metabolomic insights into bud-to-leaf development and metabolite synthesis in tea plant**

Xuecheng Zhao ^1, †^, Xiaoying Xu ^1, †^, Ning Chi^3^, Yiming Liu^1^, Xinxin Zhou^4^, Jiqiang Jin^1^, Chunlei Ma^1^, Jianqiang Ma^1^, Wei Chen^2^, Mingzhe Yao^1^* & Liang Chen^1^*

**1. Paraffin sectioning**

Tissue samples were fixed in 5-10x fixative at room temperature for ≥24 h. Dehydration used graded alcohols: 75% (4 h), 85% (2 h), 90% (1.5 h), 95% (1.5 h), and 100% (two 30-60 min steps). Samples were then cleared in alcohol-benzene (10-20 min) and xylene (two 10-20 min washes), followed by three 1-2 h paraffin infiltrations. Blocks were trimmed, cooled at -20°C, and sectioned (4 µm). Sections were floated on 40°C water, mounted on slides, dried at 60°C, and stored at room temperature.

**2.** **Plant single cell extraction method**

Leaf samples were flash-frozen in liquid nitrogen and ground to powder. The powder was homogenized in 5 mL Galbraith buffer (containing 1M DTT and 0.4 U/μL RNase inhibitor) using a Pasteur pipette, then filtered through a 40 μm cell strainer. After washing with 2 mL buffer and centrifugation (500 g, 5 min, 4°C), the pellet was resuspended in 1 mL washing buffer and stained with 50 μg/mL PI.

Nuclei were isolated using a BD FACSAria III sorter (70 μm nozzle, 4-way purity mode) into 1.5 mL tubes containing landing buffer, yielding ~50,000 nuclei per 10-min run. Nuclear integrity and concentration were assessed by phase-contrast microscopy and automated cell counting, respectively.

Galbraith, et al. Rapid flow cytometric analysis of the cell cycle in intact plant tissues. *Science*. 1983, 220: 1049-1051.

Yang, et al. Systematic methods for isolating high purity nuclei from ten important plants for omics interrogation. *Cells*. 2022, 11: 3919.

Neumann, et al. A 3D gene expression atlas of the floral meristem based on spatial reconstruction of single nucleus RNA sequencing data. *Nat. Commun*. 2022, 13: 2838.

**3. Detailed descriptions of Data Quality Control, Preprocessing**

Downstream analysis was performed using Seurat (v4.4.0) with the following workflow: First, low-quality cells (<500 detected genes) and those with >25% mitochondrial transcripts were excluded. Doublet removal was implemented via DoubletFinder (v2.0.4). The filtered dataset underwent standard preprocessing including normalization (NormalizeData), variable feature selection (FindVariableFeatures), and scaling (ScaleData). Principal component analysis (RunPCA) preceded clustering analysis (FindNeighbors/FindClusters), with results visualized through UMAP (DimPlot). For multi-batch samples, batch effects were mitigated using harmony integration, while single-batch samples proceeded without correction. Cluster-specific marker genes were identified (FindAllMarkers) and subsequently analyzed for functional enrichment using clusterProfiler (v4.12.6) with default parameters.

**4. Single-nucleus capture, library preparation and sequencing**

Live nuclei were sorted and captured for library preparation using the 10x Genomics Chromium Next GEM Single Cell 3' GEM, Library & Gel Bead Kit v3.1. Library quality was assessed using an Agilent Bioanalyzer High Sensitivity chip. Sequencing was performed on the Illumina NovaSeq 6000 platform with a paired-end 150-bp strategy.

**5. SnRNA-seq computational analysis**

Raw sequencing data were demultiplexed and converted to FASTQ format using Illumina *bcl2fastq*. Further processing, including barcode demultiplexing, gene counting, and alignment to the *Tea Plant Information Archive* genome, was performed with *CellRanger* (v7.1.0).

Quality-filtered cells (*nFeature_RNA* > 500) were analyzed in *Seurat* (v4.1.1) for dimensionality reduction (PCA), clustering, and batch correction using *Harmony* (v0.1.0). Doublets were removed with *DoubletFinder* (v2.0.3). Cell clusters were visualized via t-SNE and UMAP.

**Gene Expression Analysis**

1. Normalization: *"NormalizeData"* for gene expression quantification.
2. Variable gene selection: *"FindVariableGenes"*, followed by scaling (*"ScaleData"*).
3. PCA: *"RunPCA"* on variable genes, with batch correction via *Harmony*.
4. Clustering: *"FindClusters"* (resolution optimized).
5. Visualization: *"RunTSNE"* and *"RunUMAP"* for 2D projections.
6. Marker identification: *"FindAllMarkers"* (|avg_log2FC| > 0.26, *p_val_adj* < 0.05).
7. AUC scoring: *"FindAllMarkers"* for marker gene validation.

**6.Pseudo-time trajectory analysis**

We conducted pseudotime analysis to trace differentiation pathways using Monocle2 and Monocle3 (version 1.0.0) in accordance with established protocols. The detailed workflow can be found at <https://cole-trapnell-lab.github.io/monocle-release/docs/>.

**7. CytoTRACE**

CytoTRACE analysis was performed to assess cellular state dynamics. This computational approach reconstructs differentiation trajectories by evaluating single-cell gene expression profiles, enabling prediction of transitional states between cell populations. Using machine learning, the algorithm quantifies continuous developmental changes and characterizes cellular heterogeneity. For methodological details, refer to: <https://cytotrace.stanford.edu/>.
